# Molecular Analysis of Bacterial Isolates From Necrotic Wheat Leaf Lesions Caused by *Xanthomonas translucens*, and Description of Three Putative Novel Species, *Sphingomonas albertensis* sp. nov., *Pseudomonas triticumensis* sp. nov. and *Pseudomonas foliumensis* sp. nov.

**DOI:** 10.3389/fmicb.2021.666689

**Published:** 2021-05-19

**Authors:** James T. Tambong, Renlin Xu, Suzanne Gerdis, Greg C. Daniels, Denise Chabot, Keith Hubbard, Michael W. Harding

**Affiliations:** ^1^Ottawa Research and Development Centre, Agriculture and Agri-Food Canada, Ottawa, ON, Canada; ^2^Crop Diversification Centre South, Alberta Agriculture and Forestry, Brooks, AB, Canada

**Keywords:** wheat bacterial leaf streak disease, *Xanthomonas translucens*, cultivable bacteria, Genome-based DNA-DNA hybridization (gDDH), Average Nucleotide Identity (ANI), Multilocus sequence analysis (MLSA), MALDI-TOF

## Abstract

*Xanthomonas translucens* is the etiological agent of the wheat bacterial leaf streak (BLS) disease. The isolation of this pathogen is usually based on the Wilbrink’s-boric acid–cephalexin semi-selective medium which eliminates 90% of other bacteria, some of which might be novel species. In our study, a general purpose nutrient agar was used to isolate 49 bacterial strains including *X. translucens* from necrotic wheat leaf tissues. Maximum likelihood cluster analysis of 16S rRNA sequences grouped the strains into 10 distinct genera. *Pseudomonas* (32.7%) and *Pantoea* (28.6%) were the dominant genera while *Xanthomonas, Clavibacter* and *Curtobacterium* had 8.2%, each. *Erwinia* and *Sphingomonas* had two strains, each. BLAST and phylogenetic analyses of multilocus sequence analysis (MLSA) of specific housekeeping genes taxonomically assigned all the strains to validly described bacterial species, except three strains (10L4B, 12L4D and 32L3A) of *Pseudomonas* and two (23L3C and 15L3B) of *Sphingomonas*. Strains 10L4B and12L4D had *Pseudomonas caspiana* as their closest known type strain while strain 32L3A was closest to *Pseudomonas asturiensis*. *Sphingomonas* sp. strains 23L3C and 15L3B were closest to *S. faeni* based on MLSA analysis. Our data on MLSA, whole genome-based cluster analysis, DNA-DNA hybridization and average nucleotide identity, matrix-assisted laser desorption/ionization-time-of-flight, chemotaxonomy and phenotype affirmed that these 5 strains constitute three novel lineages and are taxonomically described in this study. We propose the names, *Sphingomonas albertensis* sp. nov. (type strain 23L3C^T^ = DOAB 1063^T^ = CECT 30248^T^ = LMG 32139^T^), *Pseudomonas triticumensis* sp. nov. (type strain 32L3A^T^ = DOAB 1067^T^ = CECT 30249^T^ = LMG 32140^T^) and *Pseudomonas foliumensis* sp. *nov.* (type strain 10L4B^T^ = DOAB 1069^T^ = CECT 30250^T^ = LMG 32142^T^). Comparative genomics of these novel species, relative to their closest type strains, revealed unique repertoires of core secretion systems and secondary metabolites/antibiotics. Also, the detection of CRISPR-Cas systems in the genomes of these novel species suggests an acquired mechanism for resistance against foreign mobile genetic elements. The results presented here revealed a cohabitation, within the BLS lesions, of diverse bacterial species, including novel lineages.

## Introduction

Members of the *Xanthomonas translucens* group are gram-negative plant pathogenic bacteria that can cause serious diseases to important cereal crops as well as forage grasses (Hersemann et al., 2017; Curland et al., 2018; Sapkota et al., 2020). The group is subdivided into 10 different pathovars based on biochemical and molecular characteristics and host ranges (van den Mooter et al., 1987; Sapkota et al., 2020) and are taxonomically placed into the translucens and graminis subgroups (Vauterin et al., 1992; Langlois et al., 2017; Peng et al., 2019). Pathovars *undulosa*, *translucens*, *horde*i, *cerealis*, *secalis* and *phleipratensis* belong to the translucens subgroup and cause bacterial leaf streak (BLS) on wheat, barley, oat, triticale, and rye (Duveiller, 1994; Vauterin et al., 1995; Sapkota et al., 2020). The “graminis” subgroup comprises of four pathovars (*arrhenatheri*, *gra*minis, *poae*, and *phlei*) that cause bacterial wilt diseases on forage grasses (Vauterin et al., 1995; Sapkota et al., 2020). Giblot-Ducray et al. (2009) published a new pathovar, *pistaciae*, that is pathogenic to pistachio and has not yet been classified to either subgroups.

Wheat BLS caused by *X. translucens* pv. *undulosa* is an important disease worldwide (Duveiller, 1990; Duveiller et al., 1997; Adhikari et al., 2012). In the last decade, the incidence of BLS in wheat has significantly increased in the Midwestern United States, the major wheat-growing regions (McMullen and Adhikari, 2011; Adhikari et al., 2012), and wheat-belt of western Canada^1^. The typical symptoms of BLS start with water-soaked streaks that subsequently become translucent necrotic leaf lesions (Smith et al., 1919; Duveiller et al., 1997; Sapkota et al., 2020), and under high disease pressure, the entire wheat leaf might be severely affected. Bacterial ooze can be seen on the leaf surface under humid and warm weather conditions. Traditional methods of isolation of the pathogen use a Wilbrink’s-boric acid-cephalexin (WBC) agar, a semi-selective medium, to eliminate other microbes (Duveiller, 1994). Duveiller (Duveiller, 1994) indicated the elimination of over 90% of “saprophytic” bacteria from washes of wheat and triticale lots using WBC, facilitating the identification of the pale yellow *X. translucens* pv. *undulosa*. It is clear that the main goal of the plant pathologist is to isolate the BLS causal agent for downstream studies such as accurate identification using biochemical, morphological and genomic evaluation, and enhancement of the biological collection as well as verification of Koch’s postulate. However, in so doing other bacterial species are eliminated, some of which might be novel genotypes.

Epi- and endo-phytic phyllosphere are habitats to millions of pathogenic and beneficial microorganisms that colonize leaves. Culture-dependent (Yashiro et al., 2011; Anguita-Maeso et al., 2019) and culture-independent methods (Grady et al., 2019; Singh et al., 2019; Regalado et al., 2020) have been used extensively to study the microbial diversity of healthy phyllosphere. Until now, however, the phylogenetic positions of cultivable bacteria associated with diseased leaf lesions, beside the causal pathogens, are not well studied.

The objectives of this study were to (i) isolate *X. translucens* pv. *undulosa* and other culturable bacteria associated with typical symptoms of BLS on wheat; (ii) characterize the diversity and taxonomic positions of the strains using 16S rRNA, multilocus sequence analysis (MLSA) and genome analysis; (iii) perform *in silico* analysis of orthologous genes, secretion systems and antimicrobial secondary metabolites of potential novel strains relative to their closest known/validly described bacterial species; and (iv) describe novel bacterial species. 16S rRNA sequence analysis is a reliable taxonomic marker for genus-level classification of bacteria (Gomila et al., 2015; Tambong, 2019) while MLSA, as single individual housekeeping genes or several housekeeping genes concatenated into pseudomolecules, has been routinely used for accurate and consistent phylogenetic resolution of strains at the species-level (Konstantinidis and Tiedje, 2007; Mulet et al., 2010; Liu et al., 2012; Tambong et al., 2014; Gomila et al., 2015; Tambong, 2019). Also the advent of genome sequencing using next generation technologies and recent advances in bioinformatics tools have revolutionized bacterial classification, providing highly reliable, reproducible and cumulative datasets and databases (Thompson et al., 2013; Parks et al., 2018; Paul et al., 2019). Genome-based parameters, such as comparative genomics, *in silico* DNA-DNA hybridization and average nucleotide identity; and matrix-assisted laser desorption/ionization-time-of-flight (MALDI-TOF) as well as biochemical characterization were used to validate the MLSA results and the hypothesis that three unique lineages discovered in this study constitute putative novel species within two bacterial genera, *Pseudomonas* and *Sphingomonas*.

## Materials and Methods

### Sample Collection and Isolation of Bacteria

Wheat flag leaves exhibiting symptoms of bacterial streak were collected from four southern Alberta fields of spring wheat (CWRS-AAC Viewfield). Four leaves per field were surface sterilized for 1 min in 2% sodium hypochlorite and rinsed three times with sterile water. The isolation of the bacteria was done as previously reported (Malette et al., 2020). Briefly, 0.25 cm^2^ section of symptomatic leaf tissue was aseptically excised, bisecting at least one bacterial lesion and immersed in a 100 μl droplet of sterile water and incubated at room temperature for 5 min. The droplet was examined under a dissecting microscope to confirm bacteria oozing into a suspension. Using a 10-μl loop, the suspensions were streaked onto nutrient agar (NA) and plates were incubated at 25°C and examined at 24 and 48 h for bacterial colonies. Up to four, morphologically-unique colonies were picked from each plate onto fresh NA plates using a sterile needle and incubated for 48 h. A total of 42 cultures were sent to the Ottawa Molecular Bacteriology Laboratory of Agriculture and Agri-Food Canada for identification. After repeated streaking (Tambong et al., 2017; Tchagang et al., 2018), 49 single colonies were obtained and preserved at −80°C in LB medium with 30% glycerol (v/v) for long-term storage. Gram-reaction of the strains was determined using the 3% KOH assay (Ryu, 1940).

### DNA Extraction, PCR Amplification, and Gene Sequencing

Strains were grown overnight in nutrient broth and genomic DNA extracted using the Wizard^®^ Genomic DNA Purification Kit (Promega Corp.) following the manufacturer’s protocol. 16S rRNA gene sequence was used for genus-level identification of the strains (Lane, 1991). Strain identification to species-level was done using housekeeping genes reported in previous taxonomic studies of *Pantoea* (*leu*S, *gyrB*, *rpo*B (Tambong et al., 2014; Tambong, 2019), *Pseudomonas* (*rpo*D, *gyr*B, *rpo*B (Mulet et al., 2010; Gomila et al., 2015; Tambong et al., 2017), *Clavibacter* (*gyr*B, *ppk*A and r*ec*A; Jacques et al., 2012), *Sphingomonas* (*atp*D, *fus*A and *rpo*B; Chen, 2012; Ogier et al., 2019), *Xanthomonas* (*atp*D and *gyr*B; Fischer-Le Saux et al., 2015; Ferreira et al., 2019), and *Curtobacterium* (*atp*D and *rpo*B; Gonçalves et al., 2019; Osdaghi et al., 2020). *Erwinia* strains were characterized to species-level based on *leu*S and *rpo*B genes and primers used for the genus *Pantoea*. All PCR amplifications were performed in a TProfessional thermal cycler (Biometra GmbH i.L., Göttingen, Germany) using Titanium *Taq polymerase* (Clontech, Inc., CA, United States) as previously described (Tambong et al., 2006). Sequencing was done with the ABI BigDye Terminator chemistry v3.1 (ThermoFisher Scientific, Canada) and run on an ABI 3500xl automated sequencer (ThermoFisher).

### BLAST and Phylogenetic Analyses

The Basic Local Alignment Search Tool (BLAST; Altschul et al., 1990) program was used to query GenBank databases with the partial gene sequences obtained in this study for homology. The individual gene loci were separately aligned in SeqMan Pro ver 15.3.0 (DNASTAR) and the 5′ and 3′ ends of DNA sequences trimmed to the shortest sequence. Geneious 11.1.5 (Biomatters Ltd.) was used to concatenate corresponding gene sequences. The individual genes and concatenated pseudogene sequences were independently aligned using MUSCLE v3.8.1551 (Edgar, 2004); and modeltest-ng (Darriba et al., 2020) was implemented to identify the best-fit models of nucleotide substitution based on the Akaike’s Information Criterion (AIC). The best-fit model of each dataset is given in the phylogenetic tree captions. Maximum likelihood phylogenetic tree inference was performed using RAxML-NG (Kozlov et al., 2019) with 1,000 non-parametric bootstrap replicates to assess internal branch robustness (Felsenstein, 1985).

### *De novo* Genome Sequencing and Analysis

Bacterial sample preparations for *de novo* genome sequencing of 7 representative strains were performed as described previously (Malette et al., 2020). Libraries were constructed using a Nextera DNA Flex Prep Kit (Illumina) following the manufacturer’s instructions. The draft genome sequences were determined by paired-end sequencing using Illumina NextSeq technology at the Molecular Technologies Laboratory, Ottawa Research and Development Centre, Ottawa, Canada. The quality of the paired-end reads, each 150 bp in length, were checked using FastQC version 0.11.3 (Andrews, 2010) and trimmed, if required. *De novo* assemblies were performed using ABySS version 1.5.2 (Simpson et al., 2009) or Unicyler v0.4.8 (Wick et al., 2017) as implemented in PATRIC (Wattam et al., 2014) and scaffolds < 300 bp in length were discarded.

Closely-related type strain genomes were determined via the Type (Strain) Genome Server (TyGS)^2^ which implements the MASH algorithm (Ondov et al., 2016); and corresponded to the closest bacterial species determined by MLSA. The Genome BLAST Distance Phylogeny approach (GBDP) as previously described was used to compute precise distances and genome-based phylogenetic relationships were inferred under the algorithm ‘trimming’ as recommended previously (Meier-Kolthoff et al., 2013). The resulting intergenomic distances were used to infer, using FASTME 2.1.4 including SPR postprocessing (Lefort et al., 2015), balanced minimum evolution trees with branch support (100 pseudo-bootstrap values). Trees were rooted at the midpoint (Farris, 1972). Genome-based DNA-DNA hybridizations (gDDH)^3^ were calculated using the default parameters of the GGDC 2.1 (Meier-Kolthoff et al., 2013) and values compared at the recommended species-level cut-off threshold of 70%. Average nucleotide identity values (ANI), were computed with default parameters using the FASTANI tool (Jain et al., 2018) to assess the taxonomic position of strains relative to the closest taxa at a species delineation cut-off threshold of 96%. The FastANI tool fragments a given query genome into overlapping fragments of a specific size. The sized fragments are then mapped to the reference genome using Mashmap (Jain et al., 2018).

For gene content analysis, the assembled genome sequences were annotated using the NCBI Prokaryotic Genome Automatic Annotation Pipeline (PGAAP) and the output used as in put data. Identification and comparison of orthologs and orthologous gene network analysis were done using OrthoVenn2 (Xu et al., 2019). The detection of bacterial secretion systems and cas3-typing (CRISPR-associated proteins) were done using, respectively, the TXSScan and cas_finder modules of MacSyFinder (Abby et al., 2014). *In silico* searches for secondary metabolites and antibiotic profiling were done against antiSMASH database-bacterial version^4^ (Blin et al., 2019).

### MALDI-TOF MS, Fatty Acid Analysis and Phenotypic Fingerprinting

Matrix-assisted laser desorption/ionization-time-of-flight MS was used to profile whole cells according to the ethanol/formic acid extraction protocol recommended by Bruker Daltonics^5^. *Pseudomonas* strains were cultured in tryptic soy agar medium and incubated at 28°C for 24 h while *Sphingomonas* strains were grown at 20°C for 72 h in trypticase soy agar. Strains were grown, processed and profiled in duplicates. Spectral measurements, mass range of 2,000-20,000 m/z, were recorded using the Microflex L20 instrument (Bruker Daltonics, Germany) and data analyzed using FlexAnalysis and BioTyper 3.1 Explorer (Bruker Daltonics). The spectra were obtained in linear, positive ion mode according to the manufacturer’s recommended settings (Bruker Daltonik, Bremen, Germany). Each final spectrum resulted from the sum of the spectra generated at random positions to a maximum of 240 shots per spectrum. Spectra were visualized and pairwise superimposed using mMass version 5.5.0 (Niedermeyer and Strohalm, 2012). Where required, log score values were generated from the main spectra and clustering and principal component (PCA) analyses done as previously described (Maier et al., 2006).

Whole cell fatty acids of the *Pseudomonas* strains were analyzed as previously reported (Tambong et al., 2017) while that of *Sphingomonas* sp. nov. strain 23L3C was analyzed as reported by Busse et al. (2003). The extraction and analysis of fatty acid methyl esters were done according to MIDI (Sherlock Microbial Identification System, version 6.2) recommended protocol (Sasser, 1990). The Agilent 7890B gas chromatograph was used to generate the profiles and automatically identified by the MIDI TSBA 6 database.

Standard biochemical and bacteriological characterizations were performed on the potential novel strains as previously described (Tambong et al., 2017) using GEN III Microplate^TM^ (Biolog) to analyze 71 carbon sources and 23 chemical sensitivity assays including pH, salt tolerance and antibiotics as recommended by manufacturer. In addition, API ZYM galleries (bioMérieux) were used to study the strains according to manufacturer’s protocol. Catalase assay was performed as previously reported (Tambong et al., 2017) and oxidase activity was tested using strips (Millipore-Sigma). Cell morphology was investigated using scanning (SEM) and transmission (TEM) electron microscopy as previously reported (Tambong et al., 2017). SEM images were captured using Hitachi SU7000 (Hitachi, Tokyo, Japan) field emission scanning electron microscopy, as recommended by manufacturer. Biolog and API ZYM assays were performed in duplicates with similar results.

## Results

### Genus-Level Identification Based on 16S rRNA

**FIGURE 1:**
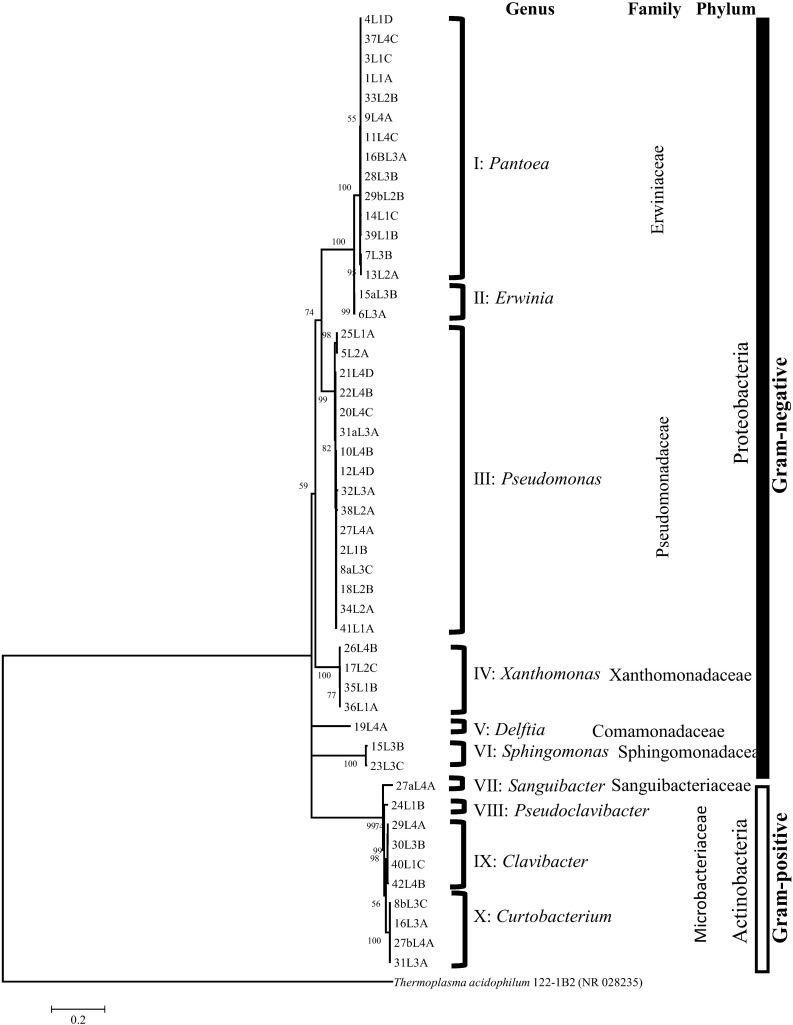
16S rRNA tree based on maximum likelihood algorithm grouped the strains into 10 clusters corresponding to distinct and validly described bacterial genera. Based on Akaaike’s information criterion, the GTR + I + G4 substitution model was used. Bootstrap values >50% are shown at the branching points.

Thirty-nine (80%) out of the 49 strains were Gram-negative bacteria while 10 strains were Gram-positive suggesting that the former major group is more abundant in the infected wheat leaf that showed typical symptoms of the bacterial leaf streak caused by *X. translucens* (Figure 1). All of the Gram-negative strains belong to the phylum Proteobacteria while the 10 Gram positive strains are of the Actinobacteria phylum. The 49 strains were taxonomically affiliated to seven bacterial families. Families *Erwiniaceae* and *Pseudomonadaceae* encompassed 65% of the strains (Figure 1). The family *Microbacteriaceae* (9 out of 10 strains) was the more abundant of the two families within the Gram-positive major group.

Forty-nine 16S rRNA sequences, l200 -1400 nt in length, were grouped phylogenetically by using the maximum likelihood algorithm into 10 unique clusters constituting different bacterial genera (Figure 1). All clusters had, at least, two strains with the exception of clusters V, VII and VIII with only one strain each (Figure 1). Comparative 16S rRNA sequence analysis using BLAST and publicly available GenBank databases taxonomically classified the strains of each of the 10 clusters into 10 valid bacterial genera. Fourteen of the 16 strains belonging to the family *Erwiniaceae* were classified as *Pantoea* (cluster I) while two strains were assigned to the genus *Erwinia* (Cluster II) (Figure 1 and Supplementary Table 1). Fourteen of the *Pantoea* strains had 16S rRNA sequence similarities in the range of 98.7 to 99.9% with *Pantoea agglomerans* DSM 3493^T^ and the other two had a sequence homology of 98.8% with *Pantoea allii* BD 390^T^ (Supplementary Table 1). Two of the *Erwiniaceae* strains (cluster II; Figure 1) exhibited 99.8% and 99.4% 16S rRNA sequence homologies to *Erwinia persicina* NBRC 102418^T^ (Supplementary Table 1). Strains of cluster III (Figure 1) belonged to the genus *Pseudomonas*; and exhibited 16S rRNA sequence similarity values in the range of 98.9 to 99.9% (Supplementary Table 1) with type strains of species with validly published names, e.g., *Pseudomonas caspiana* FB102^T^ (99.6% with strain 10L4B and 12L3D), *Pseudomonas congelans* DSM 14939^T^ (99.8% with strain 27L4A), and *Pseudomonas asturiensis* LPPA 221^T^ (99.5% with strain 32L3A) (Supplementary Table 1). Strains of cluster IV (Figure 1) were taxonomically assigned to the genus *Xanthomonas* (Family Xanthomonadaceae) exhibiting 16S rRNA similarities of 97.7% to 99.7% to *Xanthomonas translucens pv. translucens* DSM 18974^T^ (Supplementary Table 1). The 2 strains in cluster VI (Figure 1 and Supplementary Table 1) were taxonomically classified as *Sphinogomonas* (*Sphinogomonadaceae*) with 98.5% and 98.7% 16S rRNA sequence similarities to *Sphingomonas faeni* MA-olki^T^ (Supplementary Table 1). 16S rRNA nucleotide sequences of strains that are highly similar to members of the genus *Clavibacter* (*Microbacteriaceae*) were in cluster IX (Figure 1) and had similarity values of 99.3 to 99.86% (30L3B, 40L1C and 42L4B) and 99.65% (29L4A) with *Clavibacter tessellarius* ATCC 33566^T^ and *Clavibacter michiganensis* subsp. *capsici* PF008^T^, respectively (Supplementary Table 1). Four strains showing 16S rRNA nucleotide sequence homologies that are highly similar (98.6-99.4%; Supplementary Table 1) to *Curtobacterium pusillum* DSM 20527^T^ were taxonomically placed in cluster X (Figure 1). Based on 16S rRNA sequence homologies (> 98%), only one strain was identified for clusters V, VII and VIII (Figure 1); and their respective strains were classified as *Delftia* (*Comamonadaceae*), *Sanguibacter* (*Sanguibacteriaceae*) and *Pseudoclavibacter* (*Microbacteriaceae*) (Supplementary Table 1). These 3 clusters, with only strain each, were not analyzed beyond 16S rRNA.

### Species-Level Identification Based on BLAST and Phylogenetics Analyses of Housekeeping Genes

Table 1 summarizes the results of BLAST analysis of the nucleotide sequences of key taxonomic housekeeping genes used for species-level identification. Four strains exhibited 98.7% and 100.0% similarity levels with *Xanthomonas translucens* pv. *translucens* DSM 18974^T^ and *Xanthomonas translucens* pv. *undulosa* LMG 892^T^. (Table 1). Four *Curtobacterium* strains were identified to be putative *Curtobacterium flaccumfaciens* based on homology of *atp*D and *rpo*B gene sequences (Table 1). Three of the 4 *Clavibacter* strains were identified as putative *Clavibacter tessellarius* with *rec*A, *gyr*B and *pp*kA partial gene sequence homology values greater than 98% while strain 29L4A exhibited 97.7% (*ppk*A), 97.2% (*gyr*B) and 95.2% (*rec*A) sequence similarity values with *Clavibacter insidiosus* LMG 3663^T^ (Table 1). Nucleotide sequences of *leu*S, *gyr*B and *rpo*B (Tambong, 2019; Tambong et al., 2014) were used to delineate *Pantoea* strains into two species, *P. agglomerans* (type strain CIP 57.51^T^) and *P. allii* (type strain LMG 24248^T^) with homology values in the range of 98.0 to 99.0%. Fourteen strains exhibited *leu*S gene similarities of 98.9 to 100% with *P. agglomerans* CIP 57.51^T^ (= DSM 3493^T^) while two strains had percent homologies of 98.4 and 100% with *Pantoea allii* LMG 24248^T^ (Table 1). *leu*S and *rpo*B gene sequences were used to reliably and taxonomically associate the two *Erwinia* strains (6L3B and 15aL3B) with *Erwinia persicina* NBRC 102418^T^ at percent similarity values of 99.6 and 100%, respectively (Table 1).

**TABLE 1:** Species-level identification of 46 bacterial isolates obtained in this study using housekeeping gene sequence homology to publicly available GenBank sequence entries.^a^

		Percent homology of housekeeping gene sequences (%)^b^		
		
Bacterial isolates^c^	Best GenBank homologous sequence	1	2	3
1L1A, 3L1C, 4L1D, 9L4A, 11L4C, 14L1C, 16BL3A, 28L3B, 29bL4A, 33L2B, 37L4C and 39L1B	*Pantoea agglomerans* CIP 57.51^T^ (= LMG 1286^T^; FYAZ00000000)	98.9–100	99	99
13L2A and 7L3B	*Pantoea allii* LMG 24248^T^ (NTHM00000000)	98.4–100	99	98
6L3B, 15aL3B	*Erwinia persicina* NBRC 102418^T^ (BCTN01000009)	99.6	nd	100
17L2C, 26L4B, 35L1B and 36L1A	*Xanthomonas translucens* pv. *translucens* DSM 18974^T^ (CAPJ00000000)^d^	98.7	99	nd
16L3A, 27bL4A, 31bL3A and 8bL3C	*Curtobacterium flaccumfaciens* pv. *flaccumfaciens* CFBP 3418^T^ (PUEZ00000000)	98.40–98.70	97.1–99.7	99.01
30L3B, 40L1C and 42L4B	*Clavibacter tessellarius* ATCC 33566^T^ (MZMQ00000000)	98.30–98.68	98.72	99.6
29L4A	*Clavibacter insidiosus* LMG 3663^T^ (MZMO00000000)	95.2	97.2	97.6
2L1B, 8aL3C, 18L2B, 27L4A and 34L2A	*Pseudomonas congelans* LMG 21466^T^ (= DSM 14939^T^; FNJH00000000)	98.1–98.5	98.1–99.2	98.2–99.0
38L2A	*Pseudomonas coronafaciens* pv. *porri* ICMP 8961^PT^ (LJRA00000000)	99.1	99.6	98.7
5L2A	*Pseudomonas lurida* LMG 21995^T^ (PDJB00000000)	99.5	99.1	99.1
20L4C, 21L4D, 22L4B and 31aL3A	*Pseudomonas moraviensis* DSM 16007^T^ (FN554490; FN554743)	97.1	98.1	98.1
25L1A	*Pseudomonas simiae* CCUG 50988^T^ (MDFH00000000)	100	100	100
41L1A	*Pseudomonas syringae* pv. *syringae* NCPPB 281^T^ (= ICMP 3023^T^; LJRK00000000)	99.2	99.1	99
**12L4D and 10L4B**	***Pseudomonas caspiana* FBF102^T^ (LOHF00000000)**	**95.05**	**93.1**	**96.2**
**32L3A**	***Pseudomonas asturiensis* LMG 26898^T^ (FRDA00000000)**	**95.6**	**96.6**	**93.2**
**15L3B and 23L3C**	***Sphingomonas faeni* MA-olki^T^ (QAYE00000000)**	**93.1**	**95.8**	**96.7**

*^a^Where applicable, the taxonomic classsification of gene number 1 (cut-off threshold of 97%) of all genera correlated strongly with genome-based identification.*

*^b^Pantoea and Erwinia: 1, leuS; 2, gyrB; 3, rpoB. Pseudomonas: 1, rpoD; 2, gyrB; 3, rpoB; Curtobacterium: 1, atpD; 2, ppkA; 3, rpoB; Xanthomonas: 1, atpD; 2, gyrB; Clavibacter: 1, recA; 2, gyrB; 3, ppkA; and Sphingomonas: 1, atpD; 2, fusA; 3, rpoB.*

*^c^Taxa in bold are novel genotypes. ^T^ type strain; ^PT^ pathotype.*

*^d^similar homology values with Xanthomonas transluscens pv. undolusa.*

Species level identification of *Pseudomonas* strains was achieved by BLAST analysis of *rpo*D, *gyr*B, and *rpo*B as previously indicated (Tambong et al., 2017) at a cut-off threshold of 97%. The 16 *Pseudomonas* strains were classified to 8 closest validly described species based on the sequence similarity in the range of 95.0 to 100%, 94.0 to 99.0%, and 93.0 to 100%, for *rpo*D, *rpo*B and *gyr*B, respectively (Table 1). Based on this scheme, 13 *Pseudomonas* strains were accurately assigned to 6 validly described species (Table 1). For example, five strains (2L1B, 8aL3C, 18L2B, 27L4A, and 34L2A), with sequence homology values (98.1-98.5%) higher than the species cut-off threshold, were taxonomically assigned to *Pseudomonas congelans* (Table 1). Strain 32L3A, however, had the highest gene sequence homologies of 95.9% (*rpo*D), 93.2% (*rpo*B), and 96.6% (*gyr*B), with *Pseudomonas asturiensis* LPPA 221^T^ (Table 1), with three genes showing percent homology values less than the species cut-off threshold of 97% (Gomila et al., 2015; Mulet et al., 2010). Also, two strains (10L4B and 12L4D) had low percent *gyr*B, *rpo*B and *rpo*D sequence homology values of 95.1, 93, and 93.0%, respectively, with *Pseudomonas caspiana* FBF102^T^, suggesting a potential novel genotype (Table 1). The two *Sphingomonas* strains recorded percent similarity values of 96.4, 96.4 and 92.6% for *fus*A, *rpo*B, and *atp*D genes, respectively, with strain MA-olki^T^, the type strain of *Sphingomonas faeni*. These low homology levels (<97%) suggest that these two strains (15L3B and 23L3C) could be putative novel species within the genus *Sphingomonas*.

**FIGURE 2:**
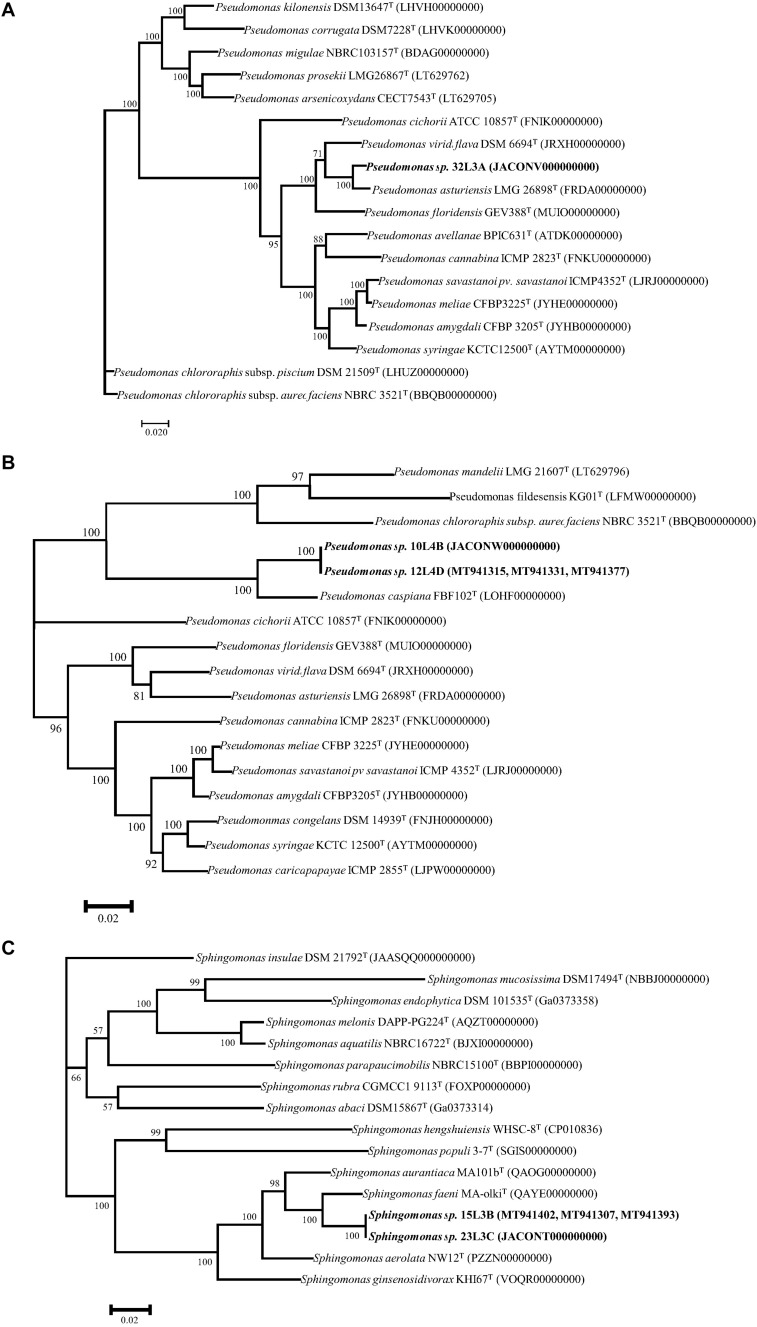
Maximum Likelihood phylogenetic trees of concatenated housekeeping gene sequences of the novel genotypes (in bold) within the genera *Pseudomonas* **(A,B)**, and *Sphingomonas* **(C)** and corresponding closest type strains. *rpo*B-*gyr*B-*rpo*D (8321 nt) and *atp*D-*fus*A-*rpo*B (6997nt) were used for *Pseudomonas*, and *Sphingomonas*, respectively. Trees were infer using RAxML-ng (Kozlov et al., 2019) with TIM2 + I + G4 **(A)** and GTR + I + G4 **(B,C)** as the best substitution models (Darriba et al., 2020) based on Akaaike’s information criterions. Bootstrap values >50% are shown at the branching points. GenBank accession numbers of closest type strains are given in parentheses.

Multilocus sequence analysis phylogenetic analyses of individual and concatenated genes were used to further characterize the taxonomic positions of these five strains (10L4B,12L4D, 32L3A, 23L3C, and 15L3B). Single gene ML evolutionary trees showed these novel strains clustering uniquely from known validly described species and well supported by 100% bootstrap values (data not shown). The ML trees (Figures 2A-C) derived from concatenated partial gene sequences specific for the respective bacterial genera (*Pseudomonas*, *rpo*B-*gyr*B-*rpo*D, 8321 nt; and *Sphingomonas*, *atp*D-*fus*A-*rpo*B, 6997nt) clustered the strains similarly to those of single gene phylogenies. These analyses re-affirmed the uniqueness of these strains and suggest that they represented three putative novel species within *Pseudomonas* and *Sphingomonas* genera.

### MALDI-TOF MS Data

**FIGURE 3:**
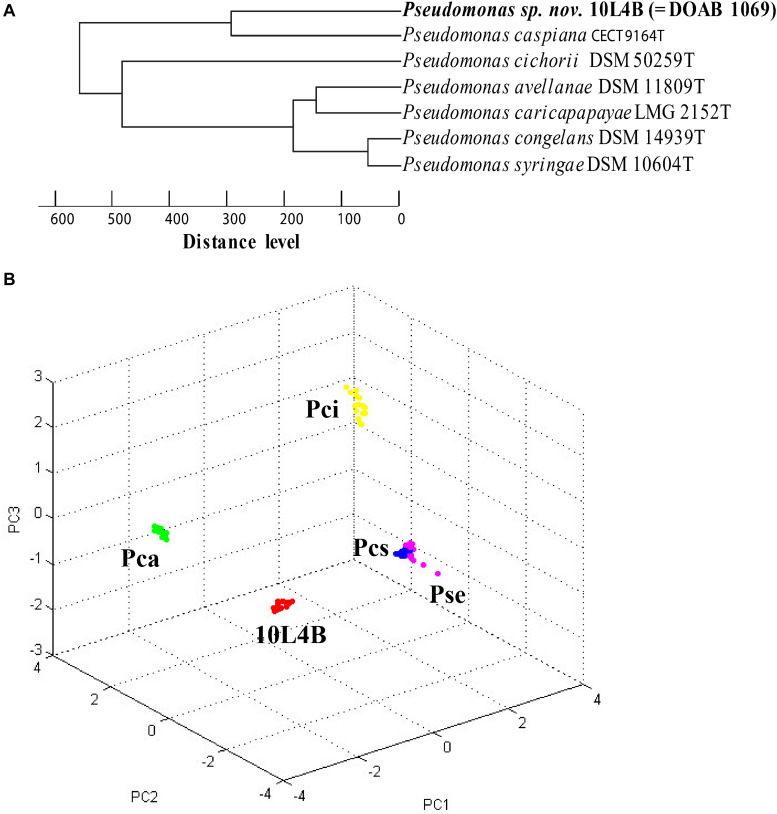
Cluster analysis of MALDI-TOF MS data of *Pseudomonas* sp. nov. 10L4B (red) compared with validly described type strains of the genus *Pseudomonas* (*P. caspiana* CEC^T^ 9164T, *P. cichorii* DSM 50259^T^, *P. avellanae* DSM 11809^T^, *P. caricapapayae* LMG 2152^T^, *P. congelans* DSM 14939^T^ and P. *syringae* DSM 106604^T^) **(A)** dendrogram, and **(B)** principal component analysis, Pca, *P. caspiana* CECT 9164^T^ (green); Pci, *P. cichorii* DSM 50259^T^(yellow); Pcs, *P. congelans* DSM 14939^T^(blue); and Pse, *P. syringae* DSM 106604^T^ (pink). Each dot represents a spectrum with 24 spectra acquired per strain.

Matrix-assisted laser desorption/ionization-time-of-flight MS is an emerging technology for quick and accurate identification of most bacterial strains by analyzing mass spectra of whole cell proteins. This technique was used to confirm that strains 32L3A, 10L4B, and 23L3C represented novel lineages relative to their corresponding closest known type strains. Pairwise comparative analysis of the spectra of the novel strains *Pseudomonas* sp. 32L3A or *Sphingomonas* sp. 23L3C and their corresponding closest known type strain revealed 8 or 7 peaks (m/z) indicated by asterisks that could be used to differentiate these strains from *P. asturiensis* or *S. faeni*, respectively (Supplementary Figure 1). For the relationship between strain 10L4B and its closest type strain (*P. caspiana*), we implemented cluster analysis to generate a dendrogram and pca (Figure 3). The novel *Pseudomonas* sp. strain 10L4B clustered distinctively at a distance level of about 250 with *Pseudomonas caspiana* CECT 9164^T^ as the closest neighbor (Figure 3A). Principal component analysis of MALDI-TOF profiles of strain 10L4B confirmed its distinct taxonomic status relative to *P. caspiana, P. cichorii, P. congelans and P. syringae* (Figure 3B). These MALDI-TOF results are consistent with MLSA-based data, suggesting that these strains represent novel *Pseudomonas* and *Sphingomonas* species.

### Genome-Based DNA-DNA Homology and Phylogenomics

To further validate strain identification, we sequenced and analyzed the genomes of 9 representative strains: 6 identified based on MLSA data to the species-level and the 3 putative novel lineages. Supplementary Table 2 shows the basic statistics of the whole genome sequences obtained in this study. All the genome sequences were of good quality. Genome size ranged from 4.1 Mb (*Sphingomonas* sp. 23L3C) to 6.0 Mb (*Pseudomonas* sp. 10L4B) with a completeness of 94.7 to 100% (Supplementary Table 2). Protein-coding genes of 3,821 (*Sphingomonas*) to 5,710 (*Pseudomonas*) (Supplementary Table 2) are consistent with the corresponding genus. Genome-based DDH (gDDH; Meier-Kolthoff et al., 2013) and average nucleotide identity (ANI; Jain et al., 2018) analyses, at the respective species cut-off threshold of 70 and 96%, validated the species-level taxonomic positions of the 6 bacterial strains identified using housekeeping genes (Table 2). For example, the genome sequence of strain 7L3B had the highest gDDH and ANI values (> threshold values of 70 and 96%, respectively) of 91.5 and 99.0%, respectively, with the type strain of *Pantoea allii* LMG 24248^T^ (NTMH00000000) while strain 1L1A was confirmed to be a putative *Pantoea agglomerans* exhibiting gDDH and ANI values of 88.6 and 98.7%, respectively, with the type strain, DSM 3493^T^ (FYAZ00000000) (Table 2). Also, the draft genome sequence of strain 17L2C had gDDH and ANI values greater than the respective threshold values with the type strain, *Xanthomonas translucens* pv. *translucens* DSM 18974^T^ (78.0 and 97.7%) and showed 94.9 and 99.7% with a reference, *Xanthomonas translucens* pv. *undulosa* Xtu 4699. This confirms that this strain belongs to pathovar *undulosa* as indicated by the pathogenic reaction on wheat and barley (data not shown). Similarly, the gDDH and ANI values of the 3 putative novel bacterial genotypes were lower than the respective species cut-off threshold levels (Table 2). For example, strain 32L3A had the highest gDDH and ANI values of 58.8 and 80.9%, respectively, with the type strain of *Pseudomonas asturiensis* LMG 26898^T^ (FRDA00000000) (Table 2). Also, strain 23L3C had only 34.4% (gDDH) and 88.8% (ANI) genome homology values with *Sphingomonas faeni* MA-olki^T^ (QAYE00000000 (Table 2). The gDDH and ANI results were corroborated by whole genome-based phylogenetic analysis inferred using GBDP-derived distances of strains 10L4B, 32L3A, and 23L3C and their corresponding closest validly described bacterial type strains (Supplementary Figure 2). For example, *Sphingomonas* sp. strain 23L3C grouped uniquely but close to *Sphingomonas faeni* MA-olki^T^ with a branch support of 100% pseudo-bootstrap values (Supplementary Figure 2). These whole genome sequence data strongly corroborated the other results and supported the hypothesis that these strains constitute putative novel genotypes within the genera *Pseudomonas* and *Sphingomonas*.

**TABLE 2:** *In silico* DNA-DNA hybridization (gDDH) and average nucleotide identity (ANI) values of *de novo* whole genome sequences of 6 representative strains identified to species level and the 3 putative novel bacterial strains^a^.

Bacterial strain	Idetification based on MultiLocus Sequence Analysis (MLSA)	gDDH (%)	ANI (%)	Closest type strain based on gDDH and ANI
7L3B	*Pantoea allii*	91.5	99.0	*Pantoea allii* LMG 24248^T^ (NTMH00000000)
1L1A	*Pantoea agglomerans*	88.6	98.7	*Pantoea agglomerans* DSM 3493^T^ (FYAZ00000000)
15aL3B	*Erwinia persicina*	95.4	99.0	*Erwinia persicina* NBRC 102418T (BCTN00000000)
17-L2C	*Xanthomonas translucens*	78.0	97.7	*Xanthomonas translucens pv. translucens* DSM 1897^T^ (CAPJ00000000)
5L2A	*Pseudomonas lurida*	98.2	99.3	*Pseudomonas lurida* DSM 15835^T^ (PDJB00000000)
25L1A	*Pseudomonas simiae*	95.5	99.4	*Pseudomonas simiae* CCUG 50988^T^ (JRMC00000000)
**32L3A**	***Pseudomonas sp.* nov.**	**58.8**	**80.9**	***Pseudomonas asturiensis* LMG 26898^T^ (FRDA00000000)**
**10L4B**	***Pseudomonas sp.* nov.**	**39.3**	**90.1**	***Pseudomonas caspiana* FBF102^T^ (LOHF00000000)**
**23L3C**	***Sphingomonas sp.* nov.**	**34.4**	**88.8**	***Sphingomonas faeni* MA-olki^T^ (QAYE00000000)**

*^a^Strains indicated in bold are novel species based on cut-off threshold values of 70.0 and 96% for gDDH and ANI respectively. Paired-end Illumina reads were generated as indicated by Malette et al. [64]. Draft genomes of Pantoea allii 7L3B (= DOAB 1050) and Pantoea agglomerans 1L1A (= DOAB1048) are from Malette et al.[64]. All the other genomes were assembled and annotated using PATRIC version 3.6.5 [107]. 17L2C = DOAB1058; 5L2A = DOAB1055; 25L1A = DOAB1064; 32L3A = DOAB1067; 10L4B = DOAB1069; 23L3C = DOAB1063; and 29L4A = DOAB1066.*

### Orthologous Gene Analysis

To provide a better insight of the genomic make-up of the proposed novel species, we analyzed the orthologous genes of the novel lineages relative to their closest type strains of species with validly published names. The gene content comparisons of the potential novel species and their closest described species indicated distinct genetic make-up.

**FIGURE 4:**
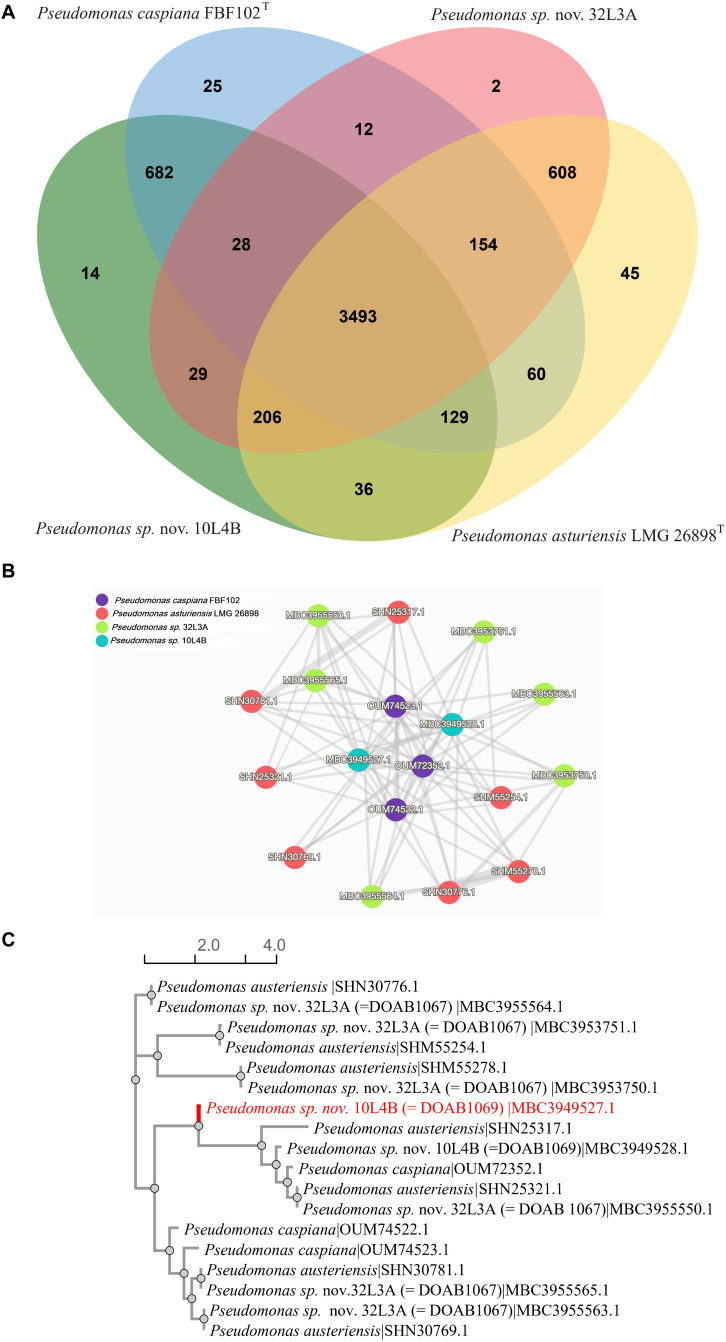
Venn diagram of the distribution of shared gene families (orthologous clusters) among the *Pseudomonas* strains **(A)**; similarity networks **(B)** and phylogenetic relationship **(C)** of the 18 protein sequences in cluster 1. Analysis was done using OrthoVenn 2 software (Xu et al., 2019).

The two potential novel *Pseudomonas* strains and their corresponding closest validly described species (*P. caspiana* and *P. asturiensis*) were analyzed together. The four whole genome sequences had a total of 18,463 protein-coding sequences. The genes were grouped into 5,523 clusters consisting of 2,061 orthologous clusters with at least two species and 3,462 single-copy gene clusters. The four *Pseudomonas* genome sequences shared 3,493 orthologous protein clusters (Figure 4A) that are involved in biological processes, molecular function and cellular component. The novel strain 10L4B shared 682 orthologous protein family clusters with its closest known type strain, *P. capsiana* FBF102^T^ while strain 32L3A shared 608 protein family clusters with *P. asturiensis* LMG 26894^T^ (Figure 4A). Strain 32L3A, however, shared only 12 protein cluster families with *P. caspiana* FBF102^T^ while strains 10L4B and *P. asturiensis* 26894^T^ uniquely shared only 36 protein family clusters. Of the 3,493 protein family clusters shared by the 4 *Pseudomonas* strains, cluster 1 had the highest protein count of 18 and is annotated as P:antibiotic biosynthetic process; IEA:UniProtKB-KW (GO:0017000; Swiss-protein hit # P0C064). The gene network of cluster 1 shows the similarity levels of *Pseudomonas* sp. nov. strain 32L3A and its closest validly described species, *P. asturiensis* and had 6 and 7 predicted protein sequences, respectively (Figure 4B). For the other pair, *Pseudomonas* sp. nov. 10L4B and its closest known bacterial relative, *P. caspiana* FBF102^T^ exhibited significantly lower numbers, 2 and 3 respectively, of this protein (Figure 4B). Protein similarity/homology was confirmed by phylogenetic analysis which revealed 5 distinct groupings, three of which are unique to *Pseudomonas* sp. 32L3A and its closest known bacterial species, *P. asturiensis* LMG 26894^T^ (Figure 4C).

The two novel species, *Pseudomonas* sp. nov. 32L3A and *Pseudomonas* sp. nov. 10L4B, had 2 and 14 unique protein family clusters, respectively that were not detected in the whole genome sequences of their respective closest type strains. The two clusters (clusters 5,279 and 5,280) of strain 32L3A had 4 proteins with no significant match to entries of the Swiss-protein databases. BLASTp analysis of the two proteins of cluster 5,279 indicates high homology to integrase, an enzyme required for integration of the phage into the host genome by site-specific recombination (Van Duyne, 2005). Cluster 5280 proteins seem to be a calcium-binding protein with 87% homology to that of *Pseudomonas corrugata* strain C8A5 isolated from the rhizosphere of painted nettles (*Plectranthus scutellarioides*).

Seven of the 14 unique protein family clusters in the novel strain 10L4B were annotated to be involved in biological processes such as ion transport (GO:0006811), nitrogen and organic acid metabolic processes. The 7 other clusters consist of 27 proteins with four protein sequences in cluster 3,534 showing low homology (42%) to BapA prefix-like domain-containing protein (WP_039297424) of *Cedecea neteri* that has been implicated in biofilm formation and host colonization (Latasa et al., 2005). The two proteins of cluster 5,286 or 5,287 were 98 or 100% similar to the demethoxyubiquinone hydroxylase family protein (WP_095100952) or SMI1/KNR4 family protein (WP_095101549), respectively in *Pseudomonas* sp. Irchel 3A5, an unclassified *Pseudomonas* strain. Protein family cluster 5,290 contains two proteins that are unique to the novel strain 10L4B. The two proteins in cluster 5,292 had about 77% homology to the suppressor of fused domain protein of *Pseudomonas* sp. Irchel 3A5 and *Pseudomonas savastanoi*. All the other clusters were annotated as hypothetical proteins and exhibited high sequence homologies (>95%) with *Pseudomonas* sp. Irchel 3A5.

**FIGURE 5:**
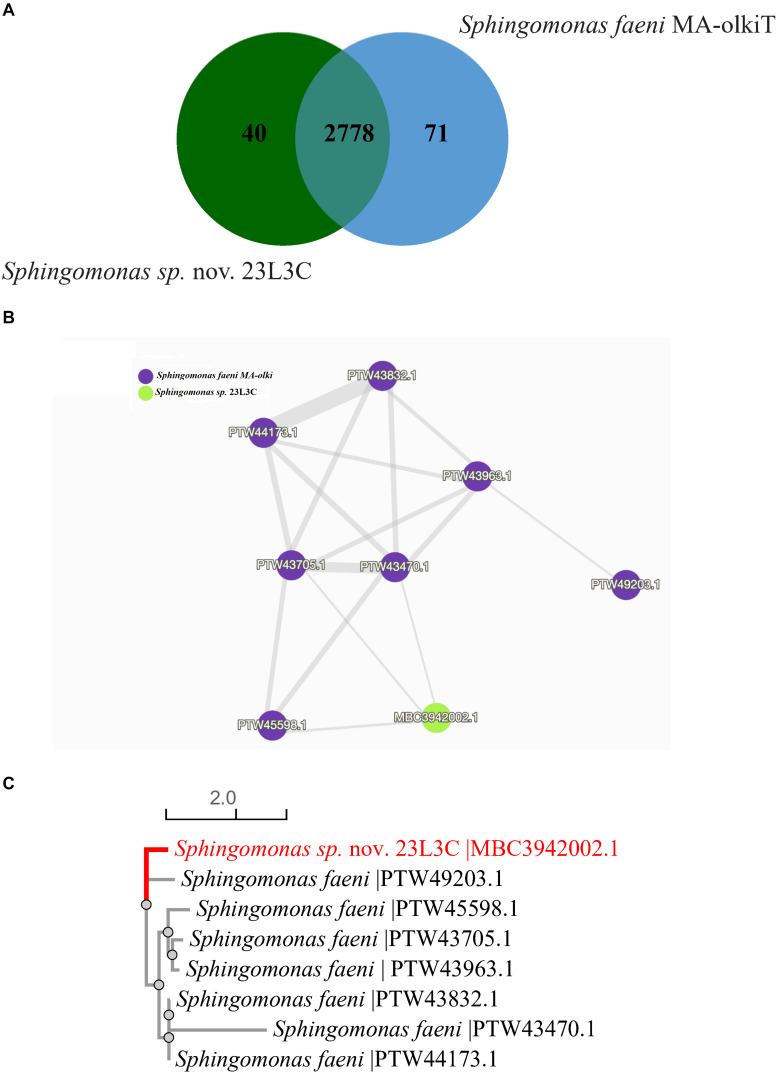
Distribution of shared gene families (orthologous clusters) between *Sphingomonas* sp. nov. 23L3C (= DOAB 1063) and *Sphingomonas faeni* MA-olki^T^ **(A)**; similarity networks **(B)** and phylogenetic relationship **(C)** of the 8 protein sequences in cluster 1. Data generated using the OrthoVenn 2 software (Xu et al., 2019).

Pairwise orthologous gene analysis for the novel *Sphingomonas* sp. strain 23L3C revealed sharing 2,778 protein family clusters with *S. faeni* MA-olki^T^, its taxonomically closest known type species; and, also, each had 40 or 71 unique protein families, respectively (Figure 5A). Cluster 1 protein family, shared by both strains, had 8 protein sequences with only one identified in the genome sequence of strain 23L3C. The protein similarity network (Figure 5B) and phylogenetic tree (Figure 5C) of cluster 1 suggest that the only protein identified in strain 23L3C is distinct. Protein sequences in cluster 1 matched the Swiss-protein # P20384, a putative transposon Tn552 DNA-invertase bin3 that facilitates horizontal transfer and recombination events related to multidrug resistance (Shore et al., 2010; Abriouel et al., 2015). This disproportionately high number (7) of protein sequences suggests that the *Sphingomonas faeni* MA-oki^T^ could be prone to acquiring antimicrobial resistance genes from the environment. Cluster 3 of the protein shared between the two strains is a family identified as a multidrug efflux pump subunit AcrB. This cluster matched entry number P31224 in the Swiss-Protein database; and seems to constitute part of the AcrA-AcrB-AcrZ-TolC complex with broad spectrum and uses the proton motive force to export substrates including antimicrobial compounds (Sennhauser et al., 2007; Hobbs et al., 2012).

Forty-one protein family clusters were identified only in the novel *Sphingomonas* sp. strain 23L3C and consisted of 89 coding sequences, ranging from 2 to 6 per cluster. Seventeen (42.5%) of the 41 family clusters could not be matched to entries in the curated Swiss-protein database. Cluster 2 showed the highest number of proteins (6) and matched entry P25184 in the Swiss-protein database with a gene ontology of P:siderophore transport, a receptor for specific transmembrane uptake of the ferric pseudobactin 358. Cluster 4 had four proteins that matched Swiss-protein number D3Z7P3, a glutaminase isoform that has been implicated in the catabolism of glutamine and plays a role in acid-base homeostasis (Cassago et al., 2012; Li et al., 2016).

A multidrug export proteins (EmrA) grouped in cluster 8 corresponded to P27303 protein of the Swiss-protein database, a part of the EMrAB-TolC tripartite efflux system that confers antibiotics resistance to compounds such as nalidixic acid and 2,4-dinitrophenol (Lomovskaya and Lewis, 1992; Borges-Walmsley et al., 2003).

### *In silico* Detection of Secondary Metabolites, and Secretion Systems

*In silico* secondary metabolite detection using AntiSMASH tool found at least three gene clusters within the genome sequence of the strains (Table 3). The types of metabolites detected included NRPS, NAGGN, acryl polyene, bacteriocin, siderophores, T3PKS, lasso peptide and terpene (Table 3). Metabolites of the aryl polylene, siderophores and bacteriocin types were more prominent in strains of *Pseudomonas* while the T3PKS type metabolites were detected only in *Sphingomonas* strains (Table 3). All the *Pseudomonas* strains have a cluster that is similar to pyoverdine with the exception of strain 10L4B (Table 3). A cluster involved in the biosynthesis of cichofactin A/cichofactin B was detected in *Pseudomonas* sp. nov. 32L3A (100% similarity) but not in its closest known species, *P. asturiensis* LMG 26898^T^. The taiwachelin biosynthetic cluster was detected in *Pseudomonas* sp. nov. 10L4B but absent in *P. caspiana* FBF102^T^ (Table 3). The novel genotype *Sphingomonas* sp. strain 23L3C had an emulsan-like cluster of the lantidin type which was not detected in the whole genome sequence of its corresponding closest known type strain, *S. faeni* Ma-olki^T^ (Table 3).

**TABLE 3:** *In silico* profiling of bacteriocin and antibiotic peptides within the genomes of the novel *Pseudomonas* and *Sphingomonas* species and their corresponding closest known type strains using the bacterial antiSMASH database^a^.

Organism	Scaffold/contig	Position	Size (kb)	Type	Most similar known cluster	Similarity (%)
*Pseudomonas sp. nov*. 32L3A	JACONV010000001	402670-469908	67.23	NRPS	Cichofactin A/cichofactin B	100
	JACONV010000005	167388-257655	90.26	NRPS	Pyoverdin	22
	JACONV010000019	28849-72448	43.60	arylpolyene	APE Vf	45
	JACONV010000020	10705-21550	10.85	bacteriocin	Merosterol	10
	JACONV010000022	67171-77201	10.03	NAGGN	-	
	JACONV010000024	1-24225	24.22	NRPS-like	Fragin	60
*P. asturiensis* LMG 26898^T^	FRDA01000001	474149-556295	82.15	terpene,NRPS	Carotenoid	100
	FRDA01000001	572607-607170	34.56	lassopeptide	-	
	FRDA01000002	217529-229379	11.85	siderophore	-	
	FRDA01000003	265834-276685	10.85	bacteriocin	-	
	FRDA01000006	234303-277902	43.60	arylpolyene	APE Vf	40
	FRDA01000017	44429-59045	14.62	NAGGN	-	
	FRDA01000018	1-35035	35.03	NRPS	Pyoverdin	6
	FRDA01000023	58327-82449	24.12	NRPS-like	Fragin	60
	FRDA01000028	1-40757	40.76	NRPS	Pyoverdin	8
*Pseudomonas sp. nov.* 10L4B	JACONW010000021	1569-52845	51.27	NRPS	Taiwachelin	33
	JACONW010000028	13622-46928	33.31	arylpolyene	APE Vf	30
	JACONW010000161	1-11572	11.57	NRPS-like	Fragin	60
	JACONW010000243	1-5228	5.23	NAGGN	-	
*P. caspiana* FBF102^T^	LOHF01000004	87603-155015	67.41	NRPS	Pyoverdin	11
	LOHF01000004	180110-194962	14.85	NAGGN	-	
	LOHF01000009	72969-83811	10.84	bacteriocin	Merosterol	10
	LOHF01000017	38234-91277	53.04	NRPS	Pyoverdin	6
	LOHF01000018	14806-58405	43.59	arylpolyene	APE Vf	45
	LOHF01000022	74296-85150	10.85	bacteriocin	-	
*Sphingomonas sp. nov.* 23L3C	JACONT010000004	36995-78074	41.08	T3PKS	-	
	JACONT010000009	23044-45352	22.31	lassopeptide	-	
	JACONT010000014	28660-70812	42.15	lanthidin	Emulsan	13
	JACONT010000020	8580-33653	25.07	terpene	Zeaxanthin	66
*Sphingomonas faeni* MA-olki^T^	QAYE01000004	183116-207667	24.55	terpene	Carotenoid	30
	QAYE01000004	254681-295745	41.06	T3PKS	-	
	QAYE01000019	1-28824	28.82	NRPS-like	-	

*^a^Blin et al. (2019); NRPS, non-ribosomal peptide synthetase cluster; NAGGAN, N-acetylglutaminylglutamine; T3PKS, type III polyketide synthase; and lassopeptide, lasso peptide.*

**FIGURE 6:**
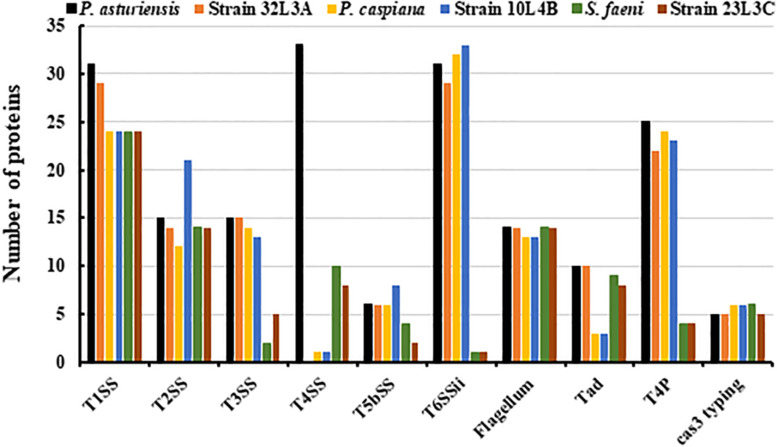
*In silico* detection of bacterial secretion systems and cas-3 sequences/genes within the genome sequences of the novel bacterial strains (*Pseudomonas* sp. nov. 10L4B (= DOAB1069); *Pseudomonas* sp. nov. 32L3A (= DOAB 1067) and *Sphingomonas* sp. nov. 23L3C (= DOAB 1063) and their respective corresponding closest validly described type strains *Pseudomonas asturiensis, Pseudomonas caspiana* and *Sphingomonas faeni.* Secretion systems- and Cas3-typing-related proteins were detected using, respectively, the TXSSCan and Cas_finder modules of MacSyFinder [1]. T1SS, T2SS, T3SS, T4SS, T5SS, and T6SS represent type I, type II, type III, type IV, type V and type VI secretion systems, respectively. Tad, Tad pili; and T4P, Type IV pili. Cas3 represents CRISPR-Cas, Clustered Regularly Interspaced Short Palindromic Repeats.

Bacterial secretion systems are essential in the virulence, pathogenicity and global cell function. We detected non-flagellar and flagellar secretion systems, showing differences between the analyzed strains (Figure 6). The presence of the T1SS, T2SS and the flagellar systems were comparable across the strains while the T3SS and T6SSii contents differed between *Pseudomonas* and *Sphingomonas* strains (Figure 6). *Sphingomonas* strains (23L3C and Ma-oki^T^) had very few genes annotated as T3SS and T6SSii, respectively (Figure 6). The novel strains 32L3A and 10L4B as well as the type strain of *P. caspiana* FBF102^T^ had very few protein coding sequences that were annotated as T4SS (Figure 6). Also, fewer counts of components of the tight adherence (Tad) pilus were detected in the novel genotype 10L4B and its corresponding closest known type strain, *P. caspiana* compared to the other strains. *Sphingomonas* strains had fewer components of the Type IV pilus (T4P) compared to the other bacterial strains studied (Figure 6) while those of the new *Pseudomonas* strains and their corresponding closest type strains were similar. Clustered Regularly Interspaced Short Palindromic Repeats (CRISPR-Cas), a defense mechanism against foreign genetic elements in prokaryotes, were detected by using MacSyFinder tool. Five or six putative and mandatory CRISPR-Cas3 type I systems were detected, with variable sequence lengths of 445-829 bp or 194-810 bp, for the *Pseudomonas* or *Sphingomonas* strains, respectively.

### Chemotaxonomic and Phenotypic Characterization

Whole cell fatty acid contents (Supplementary Table 3) of the two *Pseudomonas* novel strains were consistent with those of *sensu stricto* pseudomonads (Oyaizu and Komagata, 1983; Tambong et al., 2017). The major cellular fatty acid peaks (Supplementary Table 3) of the *Pseudomonas* strains were C_16:0,_ C_16:1_ ω6c/C_16:1_ ω7c (summed feature 3), C_18:1_ ω7c/C_18:1_ ω6c (summed feature 8), C_17:0_ cyclo; C_12:0_ 2-OH, C_12:0_ 3-OH and C_10:0_ 3-OH. Predominant fatty acid peaks in *Sphingomonas* sp. nov. 23L3C were summed feature 8 (C_18:1_ ω7c and/or C_18:1_ ω6c), summed feature 3 (C_16:1_ ω7c and/or C_16:1_ ω6c), C_14:0_ 2-OH and C_16:0_ (Supplementary Table 3). The absence of any 3-OH and the presence of C_14:0_ 2-OH fatty acids in the whole cell fatty acid content of strain 32L3A (Supplementary Table 3) is in agreement with members of the genus *Sphingomonas, sensu stricto* (Kampfer et al., 1997; Denner et al., 2001).

**FIGURE 7:**
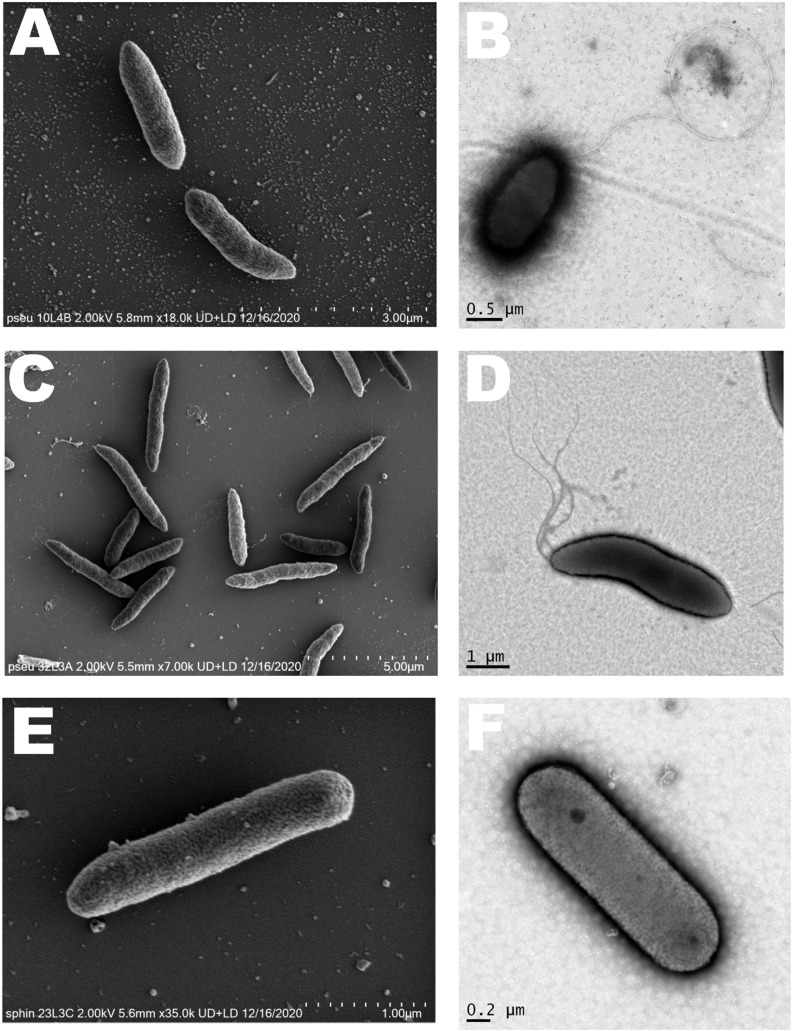
Cell morphology of the novel bacterial species. *Pseudomonas* sp. nov. strain 10L3B **(A,B)**; *Pseudomonas* sp. nov. strain 32L3A **(C,D)** and *Sphingomonas* sp. nov. strain 23L3C **(E,F)** based on scanning (left) and transmission (right) electron microscopy. Strain 10L4B has a visible polar flagellum, strain 32L3A shows multiple flagella while strain 23L3C has no visible flagellum.

Based on GENIII microplate assay (Biolog), 52, 43, or 35 carbon sources out of the 77 substrates tested were readily utilized by strain 10L4B, 32L2A or 23L3C respectively (Supplementary Table 4) and phenotypic patterns differed from those of their closest type strains. The strains were observed to be rod-shaped of variable sizes with *Sphingomonas* sp. nov. strain 23L3C being the smallest (Figure 7). Multiple polar flagella were observed on the cells of *Pseudomonas* sp. nov. strain 32L3A while strain 10L4B had one visible polar flagellum; and cells of *Sphingomonas* sp. nov. strain 23L3C did not show the presence of flagella (Figure 7).

Also, both novel *Pseudomonas* strains tolerated pH 6.0 (neutral pH) while *Sphingomonas* strain 23L3C preferred pH 5.0 (acidic condition) (Supplementary Table 4). *Pseudomonas* strains grew well at 4% salt tolerance level while the *Sphingomonas* strain did not seem to tolerate high salt concentrations (Supplementary Table 4). Both novel *Pseudomonas* strains were resistant to troleandomycin, rifamycin SV, vancomycin, lincomycin but sensitive to nalidixic acid and minocycline. Strain 23L3C is resistant to vancomycin and nalidixic acid, but sensitive to rifamycin SV, minocycline and lincomycin (Supplementary Table 4).

Using API ZYM assays, the *Pseudomonas* strains were positive for esterase (C4), esterase lipase (C 8), leucine arylamidase, valine arylamidase, cystine arylamidase, acid phosphatase, naphthol-AS-BI-phosphohydrolase, and β-glucosidase, but negative for α-glucosidase, α-galactosidases, β-galactosidases and β-galactosidases. Strain 23L3C exhibited the same enzymatic activities that were positive for the *Pseudomonas* strains but, also, uniquely exhibited high activity of β-galactosidases.

## Discussion

This study reports the analysis of cultivable bacterial strains isolated from a unique and unexploited niche, the wheat necrotic lesions caused by *Xanthomonas translucens* (the causal agent of bacterial leaf streak disease), and the identification of three novel and previously undescribed bacterial species.

Until now, research on the characteristic BLS lesions only focused on the isolation of the target pathogen for downstream studies such as accurate identification, verification of Koch’s postulate and population dynamics. To achieve this isolation objective, the Wilbrink’s-boric acid–cephalexin semi-selective medium is generally employed since it eliminates 90% of saprophytic and epiphytic bacteria (Duveiller, 1994). It is clear why plant pathologists use this semi-selective medium, but in so doing a vast amount of bacterial biodiversity is not being documented. Several other semi-selective media are routinely used for the isolation of agricultural important bacterial pathogens e.g., *Clavibacter michiganensis* on tomato seeds (Ftayeh et al., 2011) and *Pseudomonas syringae* on kiwifruit (Miyoshi and Tachibana, 1994) and thus, eliminating other bacteria, some of which might be novel taxa of unknown ecological significance.

We used a general purpose medium, nutrient agar, and isolated forty-nine bacterial strains, consisting of 10 known and validly described genera (Figure 1). The 10 bacterial genera were reliably identified to the genus-level based on 16S rRNA sequence analysis, which revealed that *Pseudomonas* (32.7%) and *Pantoea* (28.6%) were the dominant genera. Consistent with previous reports (Mulet et al., 2010; Gomila et al., 2015; Tambong et al., 2017), 16S rRNA gene sequences showed low resolution at the intrageneric level. Housekeeping genes, such as *leu*S, *atp*D, *rpo*D, *gyr*B, *fus*A, *rpo*B, *ppk*A, and *rec*A, in different combinations, have been used routinely to refine interspecfic phylogenetic and taxonomic positions of species of *Pseudomonas, Pantoea, Clavibacter, Sphingomonas* and *Curtobacterium* (Konstantinidis and Tiedje, 2007; Mulet et al., 2010; Tambong et al., 2017). *rpo*D, *rpo*B and *gyr*B are key housekeeping genes for species-level identification of *Pseudomonas* with *rpo*D reported to be the most discriminatory (Mulet et al., 2010; Gomila et al., 2015; Tambong et al., 2017) and is routinely used as the key gene for identification of species of *Pseudomonas*. *leu*S, *rpo*B and *gyr*B have been reported to provide accurate species delineation within the genus *Pantoea* (Deletoile et al., 2009; Tambong et al., 2014; Tambong, 2019). *leu*S gene fragment used in this study is a reliable marker for identification and differentiation of members of the genus *Pantoea* and is highly congruent to genome-based approach (Tambong et al., 2014; Rahimi-Khameneh et al., 2019; Tambong, 2019).

BLAST and phylogenetic (single and concatenated genes) analyses of these housekeeping genes were employed in our study and resulted in accurate species-level identification of the majority (80%) of the strains. Strains of *Pantoea* were identified to belong to either *Pantoea agglomerans* or *Pantoea allii*. *Pantoea agglomerans* is a versatile bacterium isolated from a variety of sources including wheat (Lindh et al., 1991; Kobayashi et al., 2018; Cherif-Silini et al., 2019). It was, however, surprising to isolate from wheat two strains of *Pantoea allii*, a known potent pathogen of onion (Brady et al., 2011; Rahimi-Khameneh et al., 2019). The strains of *P. allii* isolated in this study were found to be pathogenic to onion but not wheat (data not shown). Taxonomic assignment of the 4 *Xanthomonas* strains to *X. translucens* was done using *atp*D and *gyr*B, housekeeping genes that have been routinely used for differentiation of species within this genus (Parkinson et al., 2009; Fischer-Le Saux et al., 2015; Ferreira et al., 2019). Based on the *rpo*D, *rpo*B and *gyr*B (Mulet et al., 2010; Gomila et al., 2015), the *Pseudomonas* strains were accurately classified to six validly described species with the exception of five strains (32L3A, 12L4D, 10L4B, 23l3C, and 15L3B). Strain 32L3A had *Pseudomonas asturiensis* LMG 26898^T^ as its closest validly described type strain. Strains 10L4B and 12L4D were found to be identical based on high homology values (>99.0%) of their 16S rRNA, *rpo*D, *rpo*B, and *gyr*B nucleotide sequences and had *Pseudomonas caspiana* as the closest type strain. Also, the 2 strains (23L3C and 15L3B) of *Sphingomonas* were similar but taxonomically differed from their closest type strain, *Sphingomonas faeni* MA-olki^T^. As such the taxonomical positions of these three strains of *Pseudomonas* and two of *Sphingomonas* were further analyzed to determine whether they constitute novel genotypes. We used MALDI-TOF and genome-based analyses to verify the uniqueness of these bacterial strains.

Matrix-assisted laser desorption/ionization-time-of-flight MS analysis of the whole cell protein content based on spectra allowed for a straightforward separation of the novel strains from their respective closest validly described type strains. MALDI-TOF MS is an innovative tool for rapid and cost-effective bacterial identification (Bizzini and Greub, 2010; Camoez et al., 2016; Sauget et al., 2017) that has recently be integrated in clinical microbial identification workflows (Bizzini and Greub, 2010; Sauget et al., 2017). This phenotypic analysis is becoming key in rapid identification of novel bacterial species/genotypes (Moore and Rossello-Mora, 2011; Spitaels et al., 2014; Waleron et al., 2019; Lassalle et al., 2020). Even though MALDI-TOF analysis is a valuable screening tool, it might show a low discriminative power in delineating closely related bacterial species, e.g., *P. congelans* and *P. syringae*. In such cases, the corresponding results should be interpreted with caution and other methods used to validate the identification.

Rapid advances in high-throughput sequencing technologies and the development of bioinformatics tools have enabled the analysis of whole genome sequences for accurate identification, classification and taxonomy/systematics of bacterial strains (Gomila et al., 2015; Tambong, 2019). dDDH and ANI analyses based on whole genome sequence data corroborated the hypothesis that strains 32L3A, 10L4B, and 23L3C constitute novel species within the genera *Pseudomonas* and *Sphingomonas* since the computed values were lower than the cut-off threshold levels for species delineation. Genome-based techniques are reliable alternatives to wet lab DNA-DNA hybridization (wDDH) in bacterial species and strain identification (Thompson et al., 2013; Gomila et al., 2015; Parks et al., 2018; Tambong, 2019). Genome-based techniques eliminate the inherent shortcomings of wDDH such as irreproducibility between laboratories and high error (Sneath, 1989; Stackebrandt, 2003).

The availability of whole genome sequences allowed us to investigate the gene content of the novel species relative to their respective closest type strains. Analysis of several clusters of orthologous proteins showed similarities and differences. In the *Pseudomonas*, for example, all the strains had protein sequences in cluster 1 but the number of proteins was significantly different (Figure 4). The novel strain 32L3A and its corresponding type strain, *Pseudomonas asturiensis*, had 13 of the 18 protein sequences in cluster 1 while the novel strain 10L4B and its corresponding type strain (*Pseudomonas caspiana*) only had 3 and 2 proteins, respectively. This protein cluster is involved in the pathway for gramicidin S biosynthesis, an antimicrobial compound effective against some gram-positive, gram-negative bacteria and fungi (Gause and Brazhnikova, 1944; Kondejewski et al., 1996). The presence of this protein family cluster in all the *Pseudomonas* strains suggests a potential role in ecological fitness of these bacteria, especially in nutrient-restrictive niches. Still to be investigated is whether the high number of genes of this protein cluster family in *Pseudomonas* sp. nov. strain 32L3A and its corresponding closest known type strain could translate to higher competitiveness/fitness in their unique niches.

*In silico* analysis of secondary metabolites using AntiSMASH detected the presence of a pyoverdine cluster in all the genome sequences of *Pseudomonas* strains with the exception of strain 10L4B (Table 3). The lack of pyoverdine in the whole genome sequence of strain 10L4B could explain why this novel strain, when grown on King’s B medium, is non-fluorescent under ultra-voilet. Pyoverdin is a powerful iron-chelating and -transporting siderophore, and represents a ready marker for identification of some *Pseudomonas* (Meyer, 2000; Bodilis et al., 2009; Cezard et al., 2015). It is a yellow-green fluorescent pigment present in fluorescent *Pseudomonas* species (Meyer, 2000; Cezard et al., 2015).

Comparative orthologous gene analysis of the whole genome sequences of *Sphingomonas* sp. 23L3C and *S. faeni* Ma-oki^T^ identified 89 unique protein coding sequences in the former strain only. While about 42.5% of the 41 protein family clusters could not be matched to entries in the curated Swiss-protein database, three of the annotated clusters provided insight to how strain 23L3C could be competitive in the environment. Cluster 2 was annotated as a receptor for specific transmembrane uptake of ferric pseudobactin 358. BLAST analysis suggests that this could be similar to *pup*B gene, an inducible ferric-pseudobactin receptor of *Pseudomonas putida* WCS358 (Koster et al., 1993). This PupB receptor is reported to facilitate iron transport using two (pseudobactin BN7 and pseudobactin BN8) distinct heterologous siderophores (Koster et al., 1993). The presence of the ferric-pseudobactin receptor suggests that strain 23L3C could be competitive in iron-limiting environments. Also, protein family cluster 8 matched a multidrug export proteins, EmrA (P27303), a part of EMrAB-TolC tripartite efflux system that confers antibiotics resistance to compounds such as nalidixic acid and 2,4-dinitrophenol (Lomovskaya and Lewis, 1992; Borges-Walmsley et al., 2003). This could explain why, in antibiotic sensitivity studies carry out in our study, the growth of strain 23L3C was not inhibited by nalidixic acid.

Sixteen bacterial species were isolated from necrotic tissues of wheat caused by *Xanthomonas translucens* including three previously undescribed taxa (Table 1). With the exception of the undescribed taxa, *Pseudomonas lurida*, *Pseudomonas moraviensis*, and *Pseudomonas simiae*, all the other isolated species have been reported to cause diseases on field crops including wheat with distinct and characteristic symptoms. However, the only characteristic symptoms observed during sample collection were those that are specific to *Xanthomonas translucens*, the causal agent of the bacterial leaf streak disease of wheat. Preliminary pathogenicity results using some of the other known plant pathogenic bacterial species did not induce compatible reaction on wheat seedlings (data not shown). Also, the presence of some of these other bacterial species on plant leaves is not surprising. For example, *Pseudomonas lurida* (Behrendt et al., 2007) and *P. congelans* (Behrendt et al., 2003) were initially isolated from phyllosphere of healthy grasses. These arguments suggest that these other bacterial species are either saprophytes, epiphytes, or endophytes. Further work is required to elucidate the ecological role(s) of these other species.

As a taxonomic conclusion, this study isolated 49 bacterial strains from the BLS lesions on wheat using a general purpose medium, nutrient agar, instead of the frequently used Wilbrink’s-boric acid-cephalexin semi-selective medium. All the strains were identified to the species-level with the exception of these five strains: 32L3A, 10L4B, 12L4D, 15L3B and 23L3C. Polyphasic classification of these strains determined that the first three strains are members of the genus *Pseudomonas* while the latter strains belonged to *Sphingomonas sensu stricto* (Takeuchi et al., 2001). Analyses of the 16S rDNA, multilocus gene sequences, genome-based DNA–DNA hybridization and average nucleotide identity values, MALDI-TOF analysis and electron microscopy as well as biochemical/physiological traits separated these strains from their closest validly described *Pseudomonas* or *Sphingomonas* species. Based on the data from these genotypic and phenotypic analyses presented here, it is concluded that these isolates represent three novel species. We propose the names *Pseudomonas triticumensis* sp. *nov.* for strain 32L3A^T^; *Pseudomonas foliumensis* sp. *nov*. for strains 10L4B^T^ and 12L4D, and *Sphingomonas albertensis* sp. *nov.* for strains 23L3C^T^ and 15L3B.

### Description of *Pseudomonas triticumensis* sp. *nov*.

*Pseudomonas triticumensis* (tri.ti.cum.en’sis N.L. fem. adj., triticumensis from or originating from wheat, the plant from which strain 32L3A^T^ (= DOAB 1067^T^ = CECT 30249^T^ = LMG 32140^T^) was isolated.

Cells are aerobic, Gram-reaction-negative, non-spore-forming rods (0.6–1.0 μm wide and 2.0 – 4.5 μm long), motile with one, or multiple polar flagella. After 48h on KB, colonies are white-yellowish and circular (average 2-4 mm, in diameter), convex with regular margins and produce fluorescent pigments. Growth in different NaCl concentrations optimal at 4%, and grows at 4°C with optimal growth at 28-30°C and no growth at 40°C. The major cellular fatty acid peaks of the bacteria were C_16:0,_ C_16:1_ ω6c/C_16:1_ ω7c (summed feature 3), C_18:1_ ω7c/C_18:1_ ω6c (summed feature 8), C_17:0_ cyclo; C_12:0_ 2-OH, C_12:0_ 3-OH and C_10:0_ 3-OH. Based on Biolog GENIII microplate assays, strain 32L3A utilized 37 carbon sources including d-melibiose, d-arabitol, α-d-glucose, stachyose, myo- inositol, d-mannitol, d-sorbitol, d-cellobiose, gentiobiose, L-serine but not L-lactic acid, citric acid, α-keto-glutaric acid, d-malic acid, l-malic acid, α-keto-butyric acid, acetoacetic acid, propionic acid, acetic acid, d-glucuronic acid, glucuronamide, mucic acid, quinic acid, d-saccharic acid. Using API ZYM assays, these bacteria are positive for alkaline phosphatase, esterase (C4), esterase lipase (C8), leucine arylamidase, valine arylamidase cystine arylamidase, acid phosphatase, Naphthol-AS-BI-phosphohydrolase, and β-glucosidase, but negative for α-glucosidase, α-galactosidases, β-galactosidases and β-glucuronidases. The most abundant fatty acids are C_16:0_, C_16:1_ω7c and/or C_16:1_ω6c (summed feature 3) and C_18:1_ω7c and/or C_18:1_ω7c (summed feature 8). These bacteria are resistant to troleandomycin, rifamycin SV, vancomycin, lincomycin but sensitive to nalidixic acid and minocycline. The type strain is 32L3A^T^ (= DOAB 1067^T^ = CECT 30249^T^ = LMG 32140^T^), isolated from necrotic wheat leaf tissues naturally infected by *Xanthomonas translucens* from Alberta, Canada. The DNA G + C content of type strain 32L3A^T^ is 59.3mol%.

### Description of *Pseudomonas foliumensis* sp. nov.

*Pseudomonas foliumensis* (fo.lium’ensis N.L. fem. adj., foliumensis originating from leaf, the wheat leaf from which strains 12L4D and 10L4B^T^ (= DOAB 1069^T^ = CECT 30250^T^ = LMG 32142^T^) were isolated.

Cells are aerobic, Gram-reaction-negative, non-spore-forming rods (0.6 - 1.0 μm wide and 1.5 – 2.5 μm long), motile with one or two polar flagella. After 48h on KB, colonies are white-yellowish and circular (average 4 mm, in diameter), convex with regular margins and do not produce fluorescent pigments. Growth in NaCl concentrations optimal at 4%, and grows at 4°C with optimal growth at 28–30°C and no growth at 40°C. Strong growth at pH 6 but very weak growth at pH 5. The major cellular fatty acid peaks of the *Pseudomonas* strains were C_16:0,_ C_16:1_ ω6c/C_16:1_ ω7c (summed feature 3), C_18:1_ ω7c/C_18:1_ ω6c (summed feature 8), C_17:0_ cyclo; C_12:0_ 2-OH, C_12:0_ 3-OH and C_10:0_ 3-OH. Based on Biolog GENIII microplate assays, these bacteria readily utilize 47 carbon sources e.g., α-d-glucose, d-mannose, gelatin, pectin, d-fructose, d-galactose, 3-methyl glucose, d-fucose, l-rhamnose, d-raffinose, D-fucose, α-d-Lactose, d-melibiose, β-methyl-d-glucoside, d-salicin, dextrin but not gentiobiose, sucrose, l-aspartic acid, l-glutamic acid, l-histidine, d-glucuronic acid, glucuronamide, mucic acid, quinic acid, citric acid, α-keto-glutaric acid, d-malic acid and l-malic acid. Using API ZYM assays, these bacteria are positive for alkaline phosphatase, esterase (C4), esterase lipase (C8), leucine arylamidase, valine arylamidase cystine arylamidase, acid phosphatase, naphthol-AS-BI-phosphohydrolase, and β-glucosidase, but negative for α-glucosidase, α-galactosidases, β-galactosidases and β-glucuronidases. The most abundant fatty acids are C16:0, C16:1ω7c and/or C16:1ω6c (summed feature 3) and C18:1ω7c and/or C18:1ω7c (summed feature 8). These bacteria are resistant to troleandomycin, rifamycin SV, vancomycin, lincomycin but sensitive to nalidixic acid and minocycline. The type strain is 10L4B^T^ (= DOAB 1069^T^ = CECT 30250^T^ = LMG 32142^T^), isolated from necrotic wheat leaf tissues naturally infected by *Xanthomonas translucens* from Alberta, Canada. The DNA G + C content of the type strain 10L4B^T^ is 57.2%.

### Description of *Sphingomonas albertensis* sp. *nov*.

*Sphingomonas albertensis* (al.bert.en’sis N.L. fem. adj., albertensis from or originating from Alberta, the Canadian province where strains 15L3B and 23L3C^T^ (= DOAB 1063^T^ = CECT 30248^T^ = LMG 32139^T^) were isolated. Gram-negative as determined by KOH tests. Growth is observed on LB, NA, NA yeast extract and TSA at a temperature range of 5–28°C but not at 37°C. Colonies are circular, slightly convex, opaque and orange-pigmented. Cells of these bacteria are small rods, 0⋅6–0⋅7 μm × 1⋅7–2⋅6 μm. Catalase- and oxidase-positive. Flagella or endospores not observed. Aerobic. Grew at pH 5.0 but not pH 6.0. Predominant fatty acid peaks in *Sphingomonas* sp. nov. 23L3C were summed feature 8 (C_18:1_ ω7c and/or C_18:1_ ω6c), summed feature 3 (C_16:1_ ω7c and/or C_16:1_ ω6c), C_14:0_ 2-OH and C_16:0._ These bacteria were able to utilize 29 carbon source e.g., d-maltose, d-melibiose, β-methyl-d-glucoside, d-salicin, n-acetyl-β-d-mannosamine, n-acetyl-d-galactosamine, α-d-glucose, d-mannose, l-rhamnose, inosine, d-serine, d-arabitol, d-fructose-6-PO_4_, glycyl-l-proline, l-galactonic acid lactone, glucuronamide, d-malic acid, l-malic acid; but did not assimilate gentiobiose, sucrose, d-turanose, n-acetyl-d-glucosamine, n-acetyl neuraminic acid, 3-methyl glucose, d-fucose, mucic acid, quinic acid, d-saccharic acid, and citric acid. Based on API ZYM (bioMérieux), these bacteria showed high activity for alkaline phosphatase, esterase (C4), esterase lipase (C 8), leucine arylamidase, valine arylamidase, cystine arylamidase, acid phosphatase, naphthol-AS-BI-phosphohydrolase, β-galactosidases and β-glucosidase, but negative for α-glucosidase, α-galactosidases and β-glucuronidase. These bacteria are resistant to vancomycin and nalidixic acid, but sensitive to rifamycin SV, minocycline and lincomycin.

The type strain, 23L3C^T^ (= DOAB 1063^T^ = CECT 30248^T^ = LMG 32139^T^), isolated from necrotic wheat leaf tissues naturally infected by *Xanthomonas translucens* in Alberta, Canada. The DNA G + C content of the type strain 23L3C^T^ is 65.7%.

## Data Availability Statement

The seven whole-genome sequences generated in this study have been deposited at DDBJ/ENA/GenBank under the accession numbers: JACONR000000000 (strain 17L2C = DOAB1058), JACONS000000000 (15aL3B = DOAB1061), JACONT000000000 (23L3C = DOAB1063), JACONU000000000 (25L1A = DOAB1064), JACONV000000000 (32L3A = DOAB1067), JACONW000000000 (10L4B = DOAB1069), and JACONX000000000 (5L2A = DOAB1055). The version described here are the first versions: JACONR010000000, JACONS010000000, JACONT010000000, JACONU010000000, JACONV010000000, JACONW010000000, and JACONX010000000. Also, 175 DNA sequences of 16S rRNA and 245 housekeeping genes generated in this study are deposited in GenBank database under the accession numbers: MT878368-MT878416 (16S rRNA), MT941277-MT941292 (leuS), MT941293-MT941306, MT941327-MT941342 and MT941366-MT941372 (gyrB), MT941373-MT941388, MT941343-MT941358 and MT941389-MT941393(rpoB), MT941311-MT941326 (rpoD), MT941394-MT941402. (atpD), MT941359-MT941365 (ppkA), MT941308-MT941310 (recA), and MT941307 (fusA).

## Author Contributions

JT received funding, contributed to the conception, design and data collection, and analysis and write-up. MH and GD collected the diseased leaf samples and did the isolation. RX contributed in MLSA gene sequencing. SG performed genome sequencing. DC and KH generated the electron microscopy data. All authors contributed to manuscript revision, read, and approved the submitted version.

## Conflict of Interest

The authors declare that the research was conducted in the absence of any commercial or financial relationships that could be construed as a potential conflict of interest.

## Footnotes

1 https://www.grainews.ca/features/detect-and-avoid-bacterial-leaf-streak/#post-50393

2 https://www.dsmz.de/services/online-tools/tygs

3 http://ggdc.dsmz.de

4 https://antismash.secondarymetabolites.org/#!/start

5 https://www.bruker.com

## Supplementary Material

Supplementary Figure 1 Comparative analysis of matrix-assisted laser desorption/ionization-time-of-flight mass spectrometric profiles of *Pseudomonas* sp. nov. 32L3A (= DOAB 1067) and *Sphingomonas* sp. nov. 23L3C (= DOAB 1063) and their corresponding closest phylogenetic neighbors. Asterisks denote sets of seven (m/z) or eight peaks (m/z) that could be used to differentiate the novel strains from the closest known species of the respective genera. MALDI-TOF profiles were visualized using mMass 5.5.0 (Niedermeyer and Strohalm, 2012).

Supplementary Figure 2 Phylogenetic trees inferred from nucleotide distances derived from genome sequences of representative strains using the Genome BLAST Distance Phylogeny (GBDP) approach: **(A)** *Pseudomonas* sp. 10L4B; **(B)** *Pseudomonas* sp. 32L3A; and **(C)** *Sphingomonas* sp. 23L3C The branch lengths are scaled in terms of GBDP distance formula d_5_. The numbers above branches are GBDP pseudo-bootstrap support values >50% from 100 replications, with an average branch support of 99.7%. Trees were inferred with FastME 2.1.6.1 (Farris, 1972) and rooted at the midpoint (Lefort et al., 2015).
