# Corrigendum: Molecular Analysis of Bacterial Isolates From Necrotic Wheat Leaf Lesions Caused by *Xanthomonas translucens*, and Description of Three Putative Novel Species, *Sphingomonas albertensis* sp. nov., *Pseudomonas triticumensis* sp. nov. and *Pseudomonas foliumensis* sp. nov.

**DOI:** 10.3389/fmicb.2022.924519

**Published:** 2022-05-04

**Authors:** James T. Tambong, Renlin Xu, Suzanne Gerdis, Greg C. Daniels, Denise Chabot, Keith Hubbard, Michael W. Harding

**Affiliations:** ^1^Ottawa Research and Development Centre, Agriculture and Agri-Food Canada, Ottawa, ON, Canada; ^2^Crop Diversification Centre South, Alberta Agriculture and Forestry, Brooks, AB, Canada

**Keywords:** wheat bacterial leaf streak disease, *Xanthomonas translucens*, cultivable bacteria, Genome-based DNA-DNA hybridization (gDDH), Average Nucleotide Identity (ANI), Multilocus sequence analysis (MLSA), MALDI-TOF

The naming of the two new *Pseudomonas* species as *Pseudomonas triticumensis* sp. nov. and *Pseudomonas foliumensis* sp. nov. was not in accordance with the rules of the International Code of Nomenclature of Prokaryotes (ICNP) and as such cannot be validly published. This corrigendum is prepared to change the previous names to *Pseudomonas triticifolii* sp. nov. and *Pseudomonas folii* sp. nov., respectively, based on ICNP rules.

Also, the stereoisomers of the carbon sources used are now correctly indicated with capital letters, e.g., D-melibiose, D-arabitol, D-glucose, L-malic acid … etc. instead of d-melibiose, d-arabitol, d-glucose, l-malic acid...

A correction has been made to the protologues of the two novel *Pseudomonas* species, **Description of** ***Pseudomonas triticumensis* sp. nov. and Description of** ***Pseudomonas foliumensis*** **sp. nov**:

**Description of** ***Pseudomonas triticifolii*** **sp. nov**.

*Pseudomonas triticifolii* sp. nov. (tri.ti.ci.fo'li.i. L. neut. n. triticum, wheat; L. neut. n. folium, a leaf; N.L. gen. n. triticifolii, of a wheat leaf).

Cells are aerobic, Gram-reaction-negative, non-spore-forming rods (0.6–1.0 μm wide and 2.0–4.5 μm long), motile with one, or multiple polar flagella. After 48 h on KB, colonies are white-yellowish and circular (averae 2–4 mm, in diameter), convex with regular margins and produce fluorescent pigments. Growth in different NaCl concentrations optimal at 4%, and grows at 4°C with optimal growth at 28–30°C and no growth at 40°C. The major cellular fatty acid peaks of the bacteria were C_16:0_, C_16:1_ ω6c/C_16:1_ ω7c (summed feature 3), C_18:1_ ω7c/C_18:1_ ω6c (summed feature 8), C_17:0_ cyclo; C_12:0_ 2-OH, C_12:0_ 3-OH and C_10:0_ 3-OH. Based on Biolog GENIII microplate assays, strain 32L3A utilized 37 carbon sources including D-melibiose, D-arabitol, α-D-glucose, stachyose, myo- inositol, D-mannitol, D-sorbitol, D-cellobiose, gentiobiose, L-serine but not L-lactic acid, citric acid, α-keto-glutaric acid, D-malic acid, L-malic acid, α-keto-butyric acid, acetoacetic acid, propionic acid, acetic acid, D-glucuronic acid, glucuronamide, mucic acid, quinic acid, D-saccharic acid. Using API ZYM assays, these bacteria are positive for alkaline phosphatase, esterase (C4), esterase lipase (C8), leucine arylamidase, valine arylamidase cystine arylamidase, acid phosphatase, naphthol-AS-BI-phosphohydrolase, and β-glucosidase, but negative for α-glucosidase, α-galactosidases, β-galactosidases and β-glucuronidases. The most abundant fatty acids are C_16:0_, C_16:1_ω7c and/or C_16:1_ω6c (summed feature 3) and C_18:1_ω7c and/or C_18:1_ω7c (summed feature 8). These bacteria are resistant to troleandomycin, rifamycin SV, vancomycin, lincomycin but sensitive to nalidixic acid and minocycline. The type strain is 32L3A^T^ (= DOAB 1067^T^ = CECT 30249^T^ = LMG 32140^T^), isolated from necrotic wheat leaf tissues naturally infected by *Xanthomonas translucens* from Alberta, Canada. The DNA G + C content of type strain 32L3A^T^ is 59.3 %.

**Description of** ***Pseudomonas folii*** **sp. nov**.

*Pseudomonas folii* sp. nov. (fo'li.i. L. gen. n. folii, of a leaf).

Cells are aerobic, Gram-reaction-negative, non-spore-forming rods (0.6–1.0 μm wide and 1.5–2.5 μm long), motile with one or two polar flagella. After 48 h on KB, colonies are white-yellowish and circular (average 4 mm, in diameter), convex with regular margins and do not produce fluorescent pigments. Growth in NaCl concentrations optimal at 4%, and grows at 4°C with optimal growth at 28–30°C and no growth at 40°C. Strong growth at pH 6 but very weak growth at pH 5. The major cellular fatty acid peaks of the *Pseudomonas* strains were C_16:0_, C_16:1_ ω6c/C_16:1_ ω7c (summed feature 3), C_18:1_ ω7c/C_18:1_ ω6c (summed feature 8), C_17:0_ cyclo; C_12:0_ 2-OH, C_12:0_ 3-OH and C_10:0_ 3-OH. Based on Biolog GENIII microplate assays, these bacteria readily utilize 47 carbon sources e.g., α-D-glucose, D-mannose, gelatin, pectin, D-fructose, D-galactose, 3-methyl glucose, D-fucose, L-rhamnose, D-raffinose, D-fucose, α-D-Lactose, D-melibiose, β-methyl-D-glucoside, D-salicin, dextrin but not gentiobiose, sucrose, L-aspartic acid, L-glutamic acid, L-histidine, D-glucuronic acid, glucuronamide, mucic acid, quinic acid, citric acid, α-keto-glutaric acid, D-malic acid and L-malic acid. Using API ZYM assays, these bacteria are positive for alkaline phosphatase, esterase (C4), esterase lipase (C8), leucine arylamidase, valine arylamidase cystine arylamidase, acid phosphatase, naphthol-AS-BI-phosphohydrolase, and β-glucosidase, but negative for α-glucosidase, α-galactosidases, β-galactosidases and β-glucuronidases. The most abundant fatty acids are C_16:0_, C_16:1_ω7c and/or C_16:1_ω6c (summed feature 3) and C_18:1_ω7c and/or C_18:1_ω7c (summed feature 8). These bacteria are resistant to troleandomycin, rifamycin SV, vancomycin, lincomycin but sensitive to nalidixic acid and minocycline. The type strain is 10L4B^T^ (= DOAB 1069^T^ = CECT 30250^T^ = LMG 32142^T^), isolated from necrotic wheat leaf tissues naturally infected by *Xanthomonas translucens* from Alberta, Canada. The DNA G + C content of the type strain 10L4B^T^ is 57.2%.

The authors apologize for this error and state that this does not change the scientific conclusions of the article in any way.

## Publisher's Note

All claims expressed in this article are solely those of the authors and do not necessarily represent those of their affiliated organizations, or those of the publisher, the editors and the reviewers. Any product that may be evaluated in this article, or claim that may be made by its manufacturer, is not guaranteed or endorsed by the publisher.

