# Supplementary material for: Molecular Analysis of Bacterial Isolates From Necrotic Wheat Leaf Lesions Caused by Xanthomonas translucens, and Description of Three Putative Novel Species, Sphingomonas albertensis sp. nov., Pseudomonas triticumensis sp. nov. and Pseudomonas foliumensis sp. nov
Source: Front Microbiol. 2021 May 19;12:666689. doi: 10.3389/fmicb.2021.666689 (PMC8170138; doi:10.3389/fmicb.2021.666689)
Supplement: Supplementary Figure 1 — Comparative analysis of matrix-assisted laser desorption/ionization-time-of-flight mass spectrometric profiles of Pseudomonas sp. nov. 32L3A (= DOAB 1067) and Sphingomonas sp. nov. 23L3C (= DOAB 1063) and their corresponding closest phylogenetic neighbors. Asterisks denote sets of seven (m/z) or eight peaks (m/z) that could be used to differentiate the novel strains from the closest known species of the respective genera. MALDI-TOF profiles were visualized using mMass 5.5.0 (Niedermeyer and Strohalm, 2012). [file Data_Sheet_2.PDF]

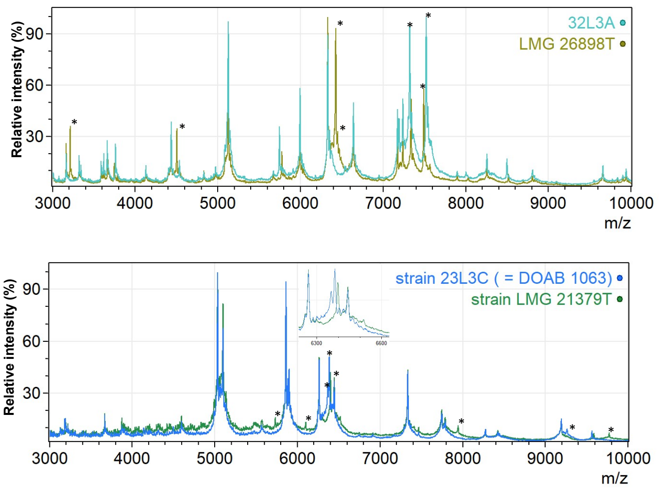

Fig. S1. Comparative analysis of matrix-assisted laser desorption/ionization-time-of-flight mass spectrometric profiles of *Pseudomonas sp.* nov. 32L3A (= DOAB 1067) **(A)** and *Sphingomonas sp.* nov. 23L3C (= DOAB 1063) and their corresponding closest phylogenetic neighbours. Asterisks denote sets of seven (m/z) or eight peaks (m/z) that could be used to differentiate the novel strains from the closest known species of the respective genera. MALDI-TOF profiles were visualized using mMass 5.5.0 (Niedermeyer and Strohalm 2012).

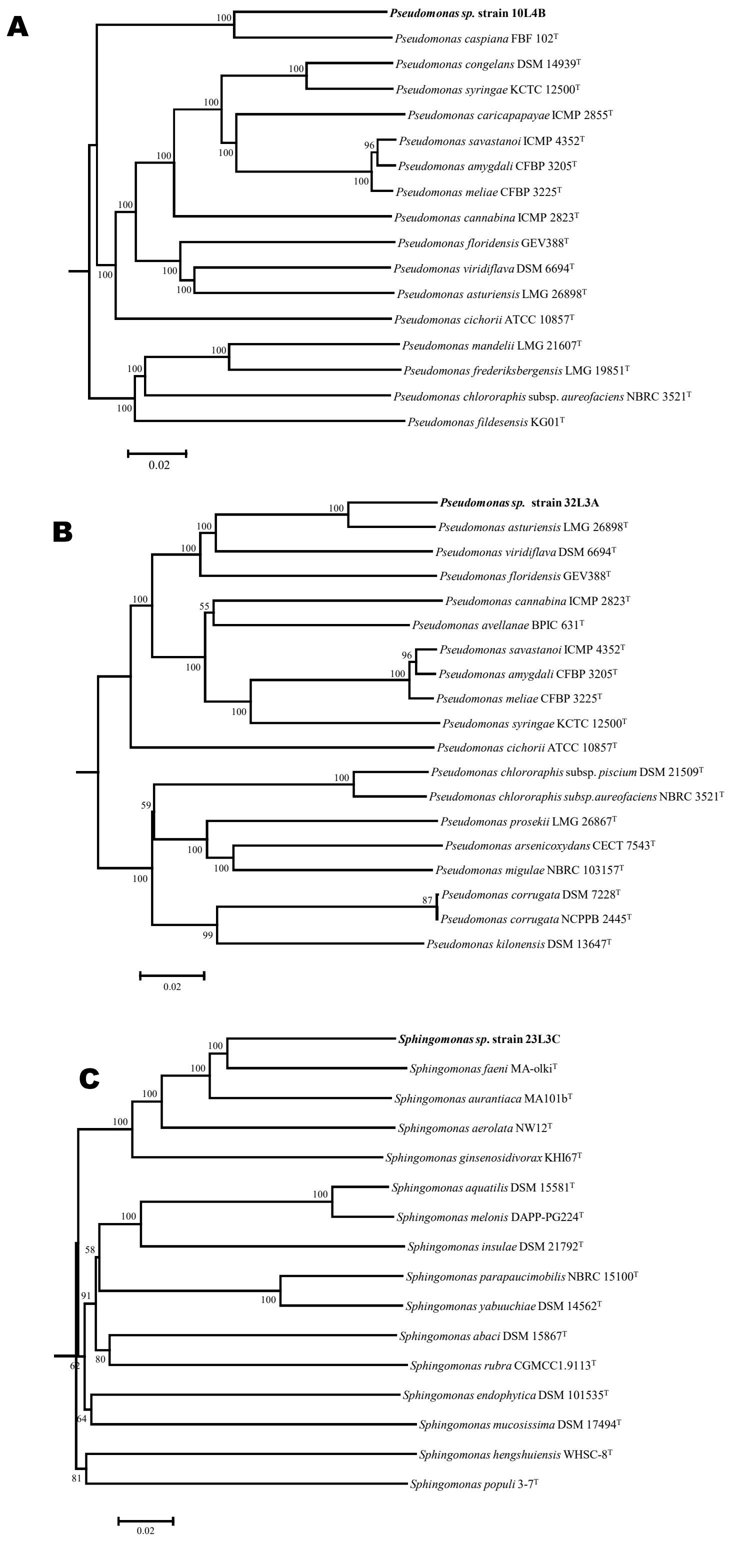

Fig. S2. Phylogenetic trees inferred from nucleotide distances derived from genome sequences of representative strains using the Genome BLAST Distance Phylogeny (GBDP) approach: (**A**) *Pseudomonas sp.* 10L4B; (**B**) *Pseudomonas sp.* 32L3A; and (**C**) *Sphingomonas sp.* 23L3C The branch lengths are scaled in terms of GBDP distance formula d_5. The numbers above branches are GBDP pseudo-bootstrap support values > 50 % from 100 replications, with an average branch support of 99.7 %. Trees were inferred with FastME 2.1.6.1 (Farris 1972) and rooted at the midpoint (Lefort et al. 2015).
