# Supplementary material for: Molecular Analysis of Bacterial Isolates From Necrotic Wheat Leaf Lesions Caused by Xanthomonas translucens, and Description of Three Putative Novel Species, Sphingomonas albertensis sp. nov., Pseudomonas triticumensis sp. nov. and Pseudomonas foliumensis sp. nov
Source: Front Microbiol. 2021 May 19;12:666689. doi: 10.3389/fmicb.2021.666689 (PMC8170138; doi:10.3389/fmicb.2021.666689)
Supplement: Supplementary file 2 [file Data_Sheet_1.PDF]

Table S1. Genus-level identification based on 16S rRNA sequence analysis of the 49 bacterial isolates obtained in this study.

Bacterial Isolates	Best 16S rRNA BLAST hit (Genus/species/strain)	GenBank accession number	Percent identity	Coverage length (bp)
1L1A	*Pantoea agglomerans* DSM 3493^T	NR_041978	99.58	1419
2L1B	*Pseudomonas syringae* pv. *coryli* NCPPB 4273^{PT}	AJ889841	99.79	1433
3L1C	*Pantoea agglomerans* DSM 3493^T	NR_041978	97.83	1243
4L1D	*Pantoea agglomerans* DSM 3493^T	NR_041978	99.06	1380
5L2A	*Pseudomonas lurida* DSM 15835^T	AJ581999	99.93	1350
6L3A	*Erwinia persicina* NBRC 102418^T	NR_114078	99.44	1418
7L3B	*Pantoea allii* BD 390^T	NR_115258	98.92	1382
8aL3C	*Pseudomonas congelans* DSM 14939^T	AJ492828	99.72	1431
8bL3C	*Curtobacterium pusillum* DSM 20527^T	NR_042315	99.41	1366
9L4A	*Pantoea agglomerans* DSM 3493^T	NR_041978	99.79	1417
10L4B	*Pseudomonas caspiana* FBF 102^T	LS483371	99.63	1342
11L4C	*Pantoea agglomerans* DSM 3493^T	NR_041978	99.64	1383
12L4D	*Pseudomonas syringae* pv. *coryli* NCPPB 4273^{PT}	LS483371	99.65	1446
13L2A	*Pantoea allii* BD 390^T	NR_115258	98.94	1413
14L1C	*Pantoea agglomerans* JCM 1236^T	AB907779	99.05	1373
15aL3B	*Erwinia persicina* NBRC 102418^T	NR_114078	99.79	1444
15L3B	*Sphingomonas faeni* MA-olki^T	NR_042129	98.49	1322
16bL3A	*Pantoea agglomerans* DSM 3493^T	NR_104943	99.71	1377
16L3A	*Curtobacterium pusillum* DSM 20527^T	LN681569	98.64	1326
17L2C	*Xanthomonas translucens* pv. *translucens* DSM 18974^T	CAJP00000000	99.64	1380
18L2B	*Pseudomonas congelans* DSM 14939^T	AJ492828	99.56	1355
19L4C	*Delftia acidovorans* JCM 5833^T	LC462156	100.00	1427
20L4C	*Pseudomonas koreensis* Ps 9-14^T	NR_025228	98.87	1421
21L4D	*Pseudomonas koreensis* Ps 9-14^T	NR_025228	98.96	1350
22L4B	*Pseudomonas koreensis* Ps 9-14^T	NR_025227	99.13	1384
23L3C	*Sphingomonas faeni* MA-olki^T	NR_042129	98.66	1344
24L1B	*Pseudoclavibacter helvolus* DSM 20419^T	NR_029264	99.63	1351
25L1A	*Pseudomonas poae* DSM 14936^T	AJ492829	99.65	1435
26L4B	*Xanthomonas translucens* pv. *translucens* DSM 18974^T	CAJP00000000	97.68	1251
27aL4A	*Sanguibacter inulinus* ST50^T	NR_029277	98.98	1373
27bL4A	*Curtobacterium pusillum* DSM 20527^T	NR_042315	99.33	1334
27L4A	*Pseudomonas congelans* DSM 14939^T	AJ492828	99.80	1385
28L3B	*Pantoea agglomerans* DSM 3493^T	NR_041978	99.71	1386
29bL4A	*Pantoea agglomerans* DSM 3493^T	NR_041978	99.72	1416
29L4A	*Clavibacter michiganensis* PF008^T	CP012573	99.65	1429
30L3B	*Clavibacter tessellarius* ATCC 33566^T	MZMQ00000000	99.64	1403
31aL3A	*Pseudomonas moraviensis* 1B4^T	NR_043314	99.03	1450
31bL3A	*Curtobacterium pusillum* DSM 20527^T	NR_042315	99.30	1437
32L3A	*Pseudomonas asturiensis* LPPA 221^T	NR_108461	99.45	1441
33L2B	*Pantoea agglomerans* DSM 3493^T	NR_041978	99.86	1445
34L2A	*Pseudomonas congelans* DSM 14939^T	AJ492828	99.57	1389
35L1B	*Xanthomonas translucens* pv. *translucens* DSM 18974^T	CAJP00000000	99.52	1459
36L1A	*Xanthomonas translucens* pv. *translucens* DSM 18974^T	CAJP00000000	99.72	1448
37L4C	*Pantoea agglomerans* DSM 3493^T	NR_041978	98.66	1396
38L2A	*Pseudomonas syringae* pv. *tomato* DC3000^R	AE016853	99.55	1341
39L1B	*Pantoea agglomerans* DSM 3493^T	NR_041978	98.59	1416
40L1C	*Clavibacter tessellarius* ATCC 33566^T	MZMQ00000000	99.86	1430
41L1A	*Pseudomonas syringae pv. coryli* NCPPB 4273^T	AJ889841	99.79	1418
42L4B	*Clavibacter tessellarius* ATCC 33566^T	MZMQ00000000	99.35	1376

^{T} Type strain; ^{PT} pathotype and ^{R} reference strain. BLAST analysis was performed as described by Altschul et al. [4].

Table S2. Basic statistics and annotations of de novo whole genome sequences of 6 representative strains identified to species level and the 3 putative novel bacterial strains[a]

Strain code	Bacterial species	Genome size (bp)	Contig count	GC (%)	N50 (bp)	N75	Contigs L50	Fine consistency (%)	Completeness (%)	Protein-Encoding Genes with Functional Assignment	Protein-Encoding Genes without Functional Assignment	Hypothetical features (%)
7L3B	*Pantoea allii*	4962883	57	53.00	312051	nd	7	94.80	100	3428	1578	21.05
1L1A	*Pantoea agglomerans*	4795525	52	55.10	147325	nd	11	95.00	100	3232	1427	19.15
15aL3B	*Erwinia persicina*	4891935	27	55.30	575033	410993	4	94.40	100	3287	1455	19.13
17-L2C	*Xanthomonas translucens*	4411941	265	68.15	32510	18646	43	96.30	100	2538	1648	32.18
5L2A	*Pseudomonas lurida*	5947209	52	60.98	230250	123803	7	92.80	100	3834	1612	18.93
25L1A	*Pseudomonas simiae*	5913872	71	60.47	165715	88578	15	91.90	96.00	3852	1584	18.95
**32L3A**	*Pseudomonas sp.*	5489169	49	59.29	232948	119803	6	94.20	99.20	3486	1529	20.10
**10L4B**	*Pseudomonas sp.*	5962777	289	57.19	35028	17695	56	93.60	100	3644	2066	26.67
**23L3C**	*Sphingomonas sp.*	4071550	78	65.72	86236	50676	15	93.30	94.70	2143	1678	36.67

[a] Paired-end Illumina reads were generated as indicated by Malette et al. [64]. Draft genomes of *Pantoea allii* 7L3B (= DOAB1050) and *Pantoea agglomerans* 1L1A (= DOAB1048) are from Malette et al.[64]. All the other genomes were assembled and annotated using PATRIC version 3.6.5 [107]. The number of Ns per 100 kbp is zero for all the draft genomes. Strains in bold are the putative novel genotypes. Strains 15aL3B = DOAB1061; 17L2C = DOAB1058; 5L2A = DOAB1055; 25L1A = DOAB1064; 32L3A = DOAB1067; 10L4B = DOAB1069; 23L3C = DOAB1063; and 29L4A = DOAB1066.

Table S3. Cellular fatty acid composition (%) of the novel species of *Pseudomonas* and *Sphingomonas*.

	Novel bacterial strain		
**Fatty acid**	**10L4B**	**32L3A**	**23L3C**
$C_{10:0}$ 3-OH	2.55	2.50	nd
$C_{12:0}$	4.46	4.49	nd
$C_{12:0}$ 2-OH	2.78	2.77	0.80
$C_{12:0}$ 3-OH	4.24	4.12	nd
$C_{14:0}$	0.28	0.21	1.82
$C_{14:0}$ 2OH	nd	nd	12.79
$C_{16:0}$	27.15	24.83	10.12
$C_{16:1}$ 2OH	nd	nd	1.31
$C_{16:0}$ w5c	0.10	nd	2.60
$C_{17:0}$	0.22	0.27	nd
$C_{17:0}$ iso	0.35	0.47	nd
$C_{17:0}$ cyclo	0.15	0.10	nd
$C_{17:1}$ w8c	0.13	0.16	0.39
$C_{17:1}$ w6c	nd	nd	1.89
$C_{18:0}$	0.80	1.63	0.30
$C_{18:1}$ 2OH	nd	nd	0.47
$C_{18:1}$ w5c	nd	nd	0.92
$C_{18:1}$ w7c 11-methyl	0.20	2.60	3.70
$C_{19:0}$ cyclo w8c	nd	nd	0.23
Summed feature 3[a]	40.59	35.65	25.97
Summed feature 8[b]	15.76	18.50	36.67

[a]$C_{16:1}$ ω7c/$C_{16:1}$ ω6c; [b]$C_{18:1}$ ω7c/$C_{18:1}$ ω6c; nd, not detected or quantities <0.1%.

Table S4. Carbon uti1ization, pH, salt tolerance, and chemical sensitivity of the three novel *Pseudomonas* and *Sphingomonas* species using GENIII Microplate™ (Biolog) showing differences relative to type strains.[a]

	**Novel bacterial strain[b]**			**Type strains**		
				P. asturiensis	*P. caspiana*	*S. faeni*
**Carbon source/chemical**	**10L4B**	**32L3A**	**23L3C**	LMG 26898	CCUG 69273	LMG 21379
**Carbon source:**						
dextrin	+	-	-	+	-	+
d-maltose	+	-	+	+	-	+
d-trehalose	+	-	-	-	-	+
d-cellobiose	+	+	-	-	-	+
gentiobiose	-	+	-	+	-	+
sucrose	-	-	-	+	-	+
d-turanose	-	-	-	+	-	±
stachyose	+	+	-	-	-	-
d-raffinose	+	+	-	+	-	-
α-d-lactose	+	+	-	+	-	+
d-melibiose	+	+	+	+	-	-
β-methyl-d-glucoside	+	-	+	-	-	-
d-salicin	+	+	+	-	-	-
N-acetyl-d-glucosamine	+	+	-	-	-	+
N-acetyl-β-d-mannosamine	+	+	+	-	-	-
N-acetyl-d-galactosamine	+	+	+	-	-	-
N-acetyl neuraminic acid	+	+	-	-	-	-
α-d-glucose	+	+	+	+	+	+
d-mannose	+	+	+	+	+	+
d-fructose	+	+	-	+	+	+
d-galactose	+	+	-	+	+	±
3-methyl glucose	+	+	-	-	-	-
d-fucose	+	-	-	-	+	-
l-fucose	-	-	-	-	-	-
l-rhamnose	+	+	+	-	±	+
inosine	+	+	+	+	+	-
d-sorbitol	+	+	-	+	+	-
d-mannitol	+	-	-	+	+	-
d-arabitol	+	+	+	+	+	-
myo-inositol	+	+	-	+	-	-
glycerol	+	+	-	+	+	-
d-glucose-6-PO_4	+	+	-	-	-	-
d-Fructose-6-PO_4	+	+	+	-	-	-
d-aspartic acid	+	+	-	-	-	-
gelatin	+	-	-	-	-	-
glycyl-l-proline	+	+	+	+	+	±
l-alanine	+	+	-	+	+	-
l-arginine	+	-	-	+	+	-
l-aspartic acid	-	-	-	+	+	±
l-glutamic acid	-	-	-	+	+	+
l-histidine	-	-	-	+	+	-
l-pyroglutamic acid	+	+	-	+	+	-
l-serine	-	+	-	+	+	-
pectin	+	-	-	+	-	+
d-galacturonic acid	+	-	-	+	+	-
l-galactonic acid Lactone	+	+	+	+	+	-

d-gluconic acid	+	-	-	+	+	+
d-glucuronic acid	-	-	-	+	+	-
glucuronamide	-	-	+	-	+	-
mucic acid	-	-	-	+	+	-
quinic acid	-	-	-	+	+	-
d-saccharic acid	+	+	-	+	+	-
p-hydroxy-phenylacetic acid	+	-	-	±	-	-
methyl pyruvate	+	+	-	+	±	-
d-lactic acid methyl ester	+	+	+	-	-	-
l-lactic acid	+	-	-	+	+	-
citric acid	-	-	-	+	+	+
α-keto-glutaric acid	-	-	-	+	+	+
d-malic acid	-	-	+	+	+	-
l-malic acid	-	-	+	+	+	+
bromo-succinic acid	-	-	+	±	-	-
tween 40	+	+	+	+	-	-
γ-amino-butryric acid	+	+	+	+	+	-
α-hydroxy-butyric acid	+	+	+	+	±	-
β-hydroxy-d,l-butyric acid	+	-	+	+	+	+
α-keto-butyric acid	-	-	+	-	±	+
acetoacetic acid	-	-	+	-	-	-
propionic acid	-	-	+	+	-	-
acetic acid	-	-	+	+	+	-
formic acid	-	-	+	+	+	-
**pH tolerance:**						
pH 6	+	+	-	+	+	+
pH 5	-	-	+	+	+	-
**Salt tolerance:**						
1% NaCl	±	±	-	+	+	+
4% NaCl	+	+	-	+	+	-
8% NaCl	-	-	-	+	+	-
**Chemical sensitivity:**						
1% Sodium Lactate	+	+	-	+	+	-
Fusidic acid	+	+	-	+	+	±
D-Serine	-	-	+	+	±	-
Troleandomycin	+	+	-	+	+	+
Rifamycin SV	+	+	-	+	+	+
Minocycline	-	-	-	+	-	-
Lincomycin	+	+	-	+	+	+
Guanidine-HCl	-	+	-	+	+	-
Niaproof 4	-	-	-	+	+	-
Vancomycin	+	+	+	+	+	±
Tetrazolium violet	+	+	+	+	+	+
Tetrazolium blue	+	+	+	+	+	+
Nalidixic acid	-	+	+	+	-	+
Lithium chloride	-	-	+	+	-	-
Potassium tellurite	-	-	+	+	+	-
Aztreonam	-	-	+	+	+	+
Sodium Butyrate	-	-	+	+	-	-
Sodium Bromate	-	-	+	+	±	-

[a] +, strain utilized the carbon source or grew in a given chemical; and -, did not utilize the carbon source or grow in the presence of a a given chemical. ±, weak growth. [b]*Pseudomonas* sp. nov. strain 10L4B; *Pseudomonas* sp. nov. strain 32L2A; and *Sphingomonas* sp. nov. strain 23L3C.
